# Substantial contribution of transported emissions to organic aerosol in Beijing

**DOI:** 10.1038/s41561-024-01493-3

**Published:** 2024-08-08

**Authors:** Kaspar R. Daellenbach, Jing Cai, Simo Hakala, Lubna Dada, Chao Yan, Wei Du, Lei Yao, Feixue Zheng, Jialiang Ma, Florian Ungeheuer, Alexander L. Vogel, Dominik Stolzenburg, Yufang Hao, Yongchun Liu, Federico Bianchi, Gaëlle Uzu, Jean-Luc Jaffrezo, Douglas R. Worsnop, Neil M. Donahue, Markku Kulmala

**Affiliations:** 1 Aerosol and Haze Laboratory, Beijing Advanced Innovation Center for Soft Matter Science and Engineering, Beijing University of Chemical Technology, Beijing, China; 2 Institute for Atmospheric and Earth System Research, Faculty of Science, University of Helsinki, Helsinki, Finland; 3 PSI Center for Energy and Environmental Sciences, Paul Scherrer Institute, Villigen, Switzerland; 4 Department of Meteorology (MISU) and Bolin Centre for Climate Research, Stockholm University, Stockholm, Sweden; 5 Nanjing-Helsinki Institute in Atmospheric and Earth System Sciences, Nanjing University, Suzhou, China; 6 Shanghai Key Laboratory of Atmospheric Particle Pollution and Prevention (LAP3), Department of Environmental Science and Engineering, Fudan University, Shanghai, China; 7 Institute for Atmospheric and Environmental Sciences, Goethe University Frankfurt, Frankfurt, Germany; 8 Institute of Materials Chemistry, TU Wien, Vienna, Austria; 9 Centre National de la Recherche Scientifique (CNRS), Institut de Recherche pour le Développement (IRD), Institute of Engineering and Management Univ. Grenoble Alpes (Grenoble INP), Institut des Géosciences de l’Environnement (IGE), Université Grenoble Alpes, Grenoble, France; 10 Aerodyne Research Inc., Billerica, MA USA; 11 Center for Atmospheric Particle Studies, Carnegie Mellon University, Pittsburgh, PA USA

**Keywords:** Atmospheric chemistry, Environmental impact, Developing world

## Abstract

Haze in Beijing is linked to atmospherically formed secondary organic aerosol, which has been shown to be particularly harmful to human health. However, the sources and formation pathways of these secondary aerosols remain largely unknown, hindering effective pollution mitigation. Here we have quantified the sources of organic aerosol via direct near-molecular observations in central Beijing. In winter, organic aerosol pollution arises mainly from fresh solid-fuel emissions and secondary organic aerosols originating from both solid-fuel combustion and aqueous processes, probably involving multiphase chemistry with aromatic compounds. The most severe haze is linked to secondary organic aerosols originating from solid-fuel combustion, transported from the Beijing–Tianjing–Hebei Plain and rural mountainous areas west of Beijing. In summer, the increased fraction of secondary organic aerosol is dominated by aromatic emissions from the Xi’an–Shanghai–Beijing region, while the contribution of biogenic emissions remains relatively small. Overall, we identify the main sources of secondary organic aerosol affecting Beijing, which clearly extend beyond the local emissions in Beijing. Our results suggest that targeting key organic precursor emission sectors regionally may be needed to effectively mitigate organic aerosol pollution.

## Abstract (web summary)

Secondary organic aerosols in Beijing are driven by emissions from outside of the city, with seasonally different emission sources, according to molecular chemical characterization of particulate air pollution.

## Main

Globally, air pollution is responsible for several million premature deaths, many of which occur where severe pollution meets a large population (in polluted megacities)^1^. In China, despite the implementation of stringent mitigation strategies^2^, a large proportion of the population is still impacted by poor air quality. A large fraction of fine particulate matter (that is, smaller than 2.5 µm, PM_2.5_) is associated with atmospherically formed secondary inorganic (SIA) and organic aerosol (SOA)^3–5^. The influence of chemical composition on PM_2.5_ health effects remains uncertain; the health risk of PM_2.5_ might not be driven by its major SIA constituents (ammonium, nitrate and sulfate), but rather on OA, dominated by SOA^6–8^. Accordingly, the recent successful reduction in SIA, especially in sulfate^9^, might not lead to the expected health benefits, and detailed knowledge about SOA sources is essential. However, the sources of SOA are uncertain, and the processes and pathways involved in its formation are not well understood^3,4,10^. Without comprehensive information, designing efficient SOA mitigation strategies remains stymied. Therefore, a detailed identification of the sources of SOA (source sector, temporal variability and spatial origin), as well as its formation processes, is essential in devising targeted effective reduction strategies.

In the atmosphere, SOA is produced by the complex processing of multiple gaseous organic compounds (for example, aromatic and biogenic). A cocktail of precursors together with atmospheric aging results in SOA with a chemical fingerprint that is similar, regardless of the original emission source, and this hinders identification of the emission sources for that SOA^11^. Widely used mass spectrometers, such as the Aerosol Chemical Speciation Monitor, fragment the measured organic molecules present in the particles, further obscuring information on the precursor molecules^12^. Only recently have newly developed field-deployable soft-ionization mass spectrometers offered semi-online characterization of OA combining substantial molecular speciation with high time resolution^13,14^ and thus greatly enhancing the potential to identify SOA sources^15–18^.

## Quantifying OA sources

**Extended Data Fig. 1:**
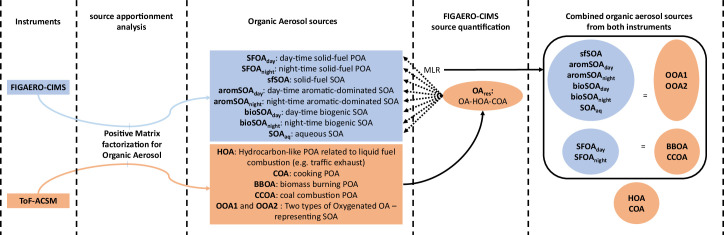
Data analysis stream used in this study. Chemical characterizations of ToF-ACSM and FIGAERO-CIMS are used to quantify the sources of primary and secondary OA. We combine the quantitative OA ToF-ACSM analyses with a factorization of time series of near-molecular organic aerosol mass spectra determined by FIGAERO-CIMS. The ToF-ACSM is used to determine the organic mass and the contribution of primary organic aerosol (POA) from liquid fossil fuel combustion (HOA) and cooking (COA); the FIGAERO-CIMS is used to produce a set of SOA sources besides POA from solid-fuel combustion, which are converted into mass loadings using a multilinear regression to the ToF-ACSM OA mass loadings, once some of the POA sources (HOA, COA) have been subtracted from it. Thereby, we obtain response factors of the FIGAERO-CIMS to the different SOA sources. Were these to be single compounds (instead of SOA sources), the response factors would be calibration factors but here they were applied across a wide range of compounds contained in a specific SOA source in this instance.

**Extended Data Fig. 2:**
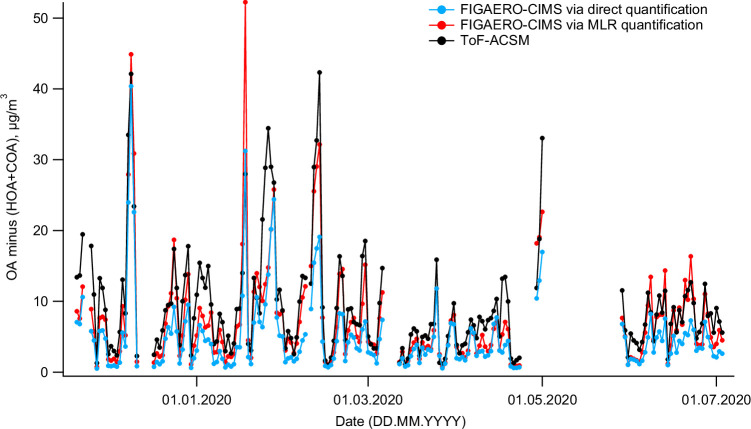
Comparison between FIGAERO-CIMS and ToF-ACSM. Daily mean concentration time series of OA minus (HOA+COA) from FIGAERO-CIMS via MLR quantification and direct quantification (see methods) and ToF-ACSM.

In this Article we use quantitative OA aerosol mass spectrometry together with high-time resolution near-molecular OA characterization to identify and quantify SOA sources and their variability in Beijing by using advanced source apportionment techniques (positive matrix factorization)^19^. We combine the quantitative OA ToF-ACSM (time-of-flight Aerosol Chemical Speciation Monitor) analyses widely used for source apportionment with a factorization of time series of near-molecular organic aerosol mass spectra determined by Filter Inlet for Gas and AEROsols coupled to an iodide Chemical Ionization Mass Spectrometer (FIGAERO-CIMS)^13^. FIGAERO-CIMS uses soft chemical ionization, which allows for the detection of molecular ions—although some are affected by thermal decomposition during the measurement—and their chemical formulae, but not their structure. Overall, the FIGAERO-CIMS can detect a wide range of different anthropogenic and biogenic OA types^20–27^. Although not all compounds in OA are detected, compared to tracer-based approaches, the FIGAERO-CIMS analyses represent a much larger OA mass fraction, estimated here to be ~61% (winter, 58%; COVID lockdown, 66%; summer, 59%), in line with previous studies^13^^,28,29^, allowing for unprecedented assessment of the main sources of SOA. We can thus use the ToF-ACSM to determine the organic mass as well as the contribution of directly emitted primary organic aerosol (POA) from combustion (HOA) and cooking (COA), and use the FIGAERO-CIMS to identify a set of SOA sources adding to POA from solid-fuel combustion. We assume that these FIGAERO-CIMS OA types constitute the ToF-ACSM OA, once HOA and COA have been accounted for, and we determine their mass loadings using multilinear regression (MLR), that is, fit the FIGAERO-CIMS OA types to OA minus (HOA + COA) from the ToF-ACSM acting as reference (Extended Data Figs. 1 and 2). To further support source identification, we rely on comparisons to laboratory SOA experiments and external tracers, for example, gas-phase oxidation products or particle-phase molecular organic source marker measurements based on PM_2.5_ filters.

**Fig. 1:**
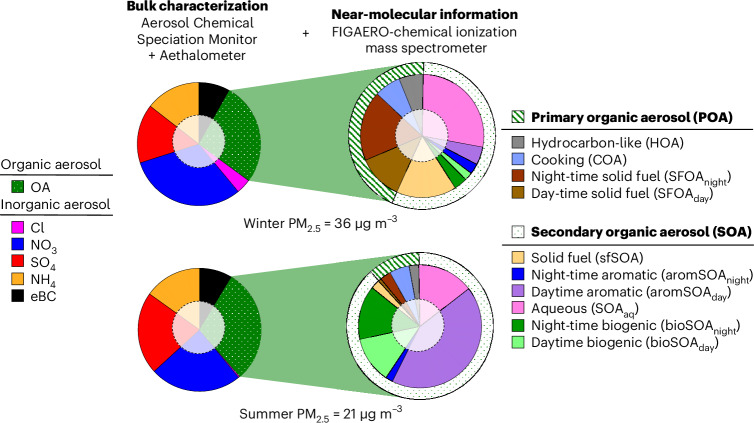
Sources of OA and their contribution to fine particle mass in winter (20 November 2019 to 25 January 2020) and summer (1 May 2020 to 2 July 2020). The bulk chemical composition of PM_2.5_ from an Aerosol Chemical Speciation Monitor and Aethalometer (ToF-ACSM and AE33, left) shows that roughly one-third of the PM_2.5_ mass is organic, without detailed information about its sources. Additional near-molecular information based on thermal desorption chemical ionization mass spectra (FIGAERO-CIMS, right) reveals that, even in winter, well over half of the OA is secondary, with a large contribution of aqueous processing. Solid-fuel sources with primary and secondary constituents comprise almost half of wintertime OA. During summertime, half of the OA is secondary organic aerosol from aromatics, probably from mobile sources, and a quarter has biogenic origin. For the COVID lockdown period (26 January 2020 to 30 April 2020) see Extended Data Fig. 3. The holes in the pie charts represent constituents and sources not covered, which comprise less than 20% of the whole. A sensitivity assessment is presented in Extended Data Figs. 4 and 5 and shows that the FIGAERO-CIMS alone directly measures ~61% of the OA mass concentration based on MLR quantification, also including HOA and COA from the ToF-ACSM, presented in this figure.

**Extended Data Fig. 3:**
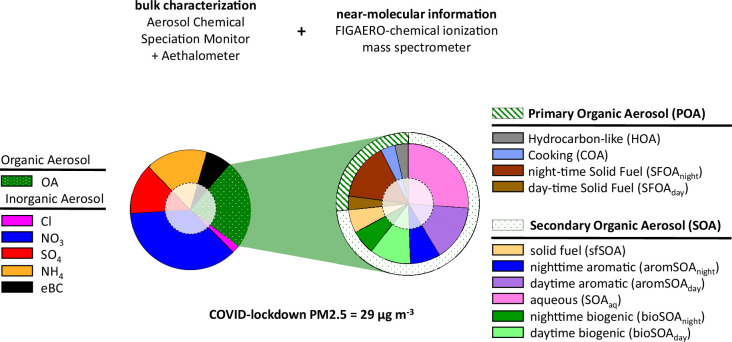
Sources of organic aerosol and their contribution to fine particle mass during the COVID lockdown period. Bulk chemical composition of particulate matter from an Aerosol Chemical Speciation Monitor and Aethalometer (ToF-ACSM and AE33, left) shows that roughly one-quarter of the particulate matter mass is organic without detailed information on the sources. Additional near-molecular information based on thermal desorption chemical ionization mass spectra (FIGAERO-CIMS, right) reveals that three-quarter of the organic aerosol is secondary, with a roughly equal contributions of biogenic SOA, aromatic-dominated-SOA, and aqueous SOA. The holes in the pie charts are constituents and sources not covered, which comprise less than 20%.

Beijing’s PM_2.5_ bulk composition is shown in Fig. 1 for a typical urban location in Beijing. Coinciding with the lunar new year in 2020 (25 January 2020), the global COVID pandemic led to a strong reduction in traffic density, coal consumption and general economic activity, all of which were restored to pre-COVID (2019) levels by the end of April 2020 (refs. ^30,31^). It is thus likely that emissions of anthropogenic PM_2.5_ and precursors were reduced during this period but largely recovered to normal levels by the end of April. Despite those emission reductions, particulate pollution levels remained high during the COVID lockdown, although at lower concentrations compared to 2019 (Extended Data Fig. 3 and Supplementary Fig. 1). We observe a clear transition from more polluted winter conditions characterized by prominent pollution episodes, with a daily PM_2.5_ mean concentration of 36 µg m^−3^, to cleaner summertime conditions, with a daily mean concentration of 21 µg m^−3^. In spite of this, the bulk chemical composition (measured by the ToF-ACSM) differs surprisingly little between seasons (Fig. 1). The PM_2.5_ was composed of 61–65% SIA, 27–30% OA and 8–9% equivalent black carbon (eBC). The SIA formed from gaseous emissions, such as NO_*x*_ and SO_2_, from fossil-fuel combustion and NH_3_ from diverse urban sources and agriculture. Throughout, the measured daily mean OA concentration was 3.3–3.5 times that of eBC, consistent with aged OA^32^ and in line with a large contribution of SOA found in previous studies^33–36^.

There are four primary OA (POA) types: HOA (hydrocarbon organic aerosol) from liquid-fossil-fuel combustion, COA (cooking OA) and SFOA (solid-fuel combustion OA), which has contributions of biomass burning OA (BBOA) and coal combustion OA (CCOA). There are also six secondary OA (SOA) types formed in the atmosphere: solid-fuel-SOA (sfSOA), aromatic-dominated-SOA (aromSOA_day_, aromSOA_night_), biogenic SOA (bioSOA_day_, bioSOA_night_) and aqueous SOA (SOA_aq_).

We find that the main sources of OA vary substantially depending on the season (Fig. 1 and Extended Data Fig. 3). In winter, less than half of the OA pollution is primary, with primary solid-fuel emissions predominating (SFOA), and much of the SOA is related to solid-fuel SOA, along with aqueous SOA produced from aqueous particle-phase chemistry. In summer, SOA predominates, and solid-fuel OA (primary or secondary) almost vanishes. Although biogenic SOA is present, it remains a relatively small contributor to OA, even in summer (in northern China). Instead, we find that SOA, in summer, is dominated by emissions from anthropogenic activities. The most prominent are aromatic emissions unrelated to solid-fuel combustion, forming almost half of the OA. Other sources, such as liquid-fuel POA (HOA; winter 6% of OA, summer 3% of OA) and cooking emissions (COA; winter 7% of OA, summer 5% of OA) contribute to a lesser extent during both seasons.

## SOA emission sources and formation

**Fig. 2:**
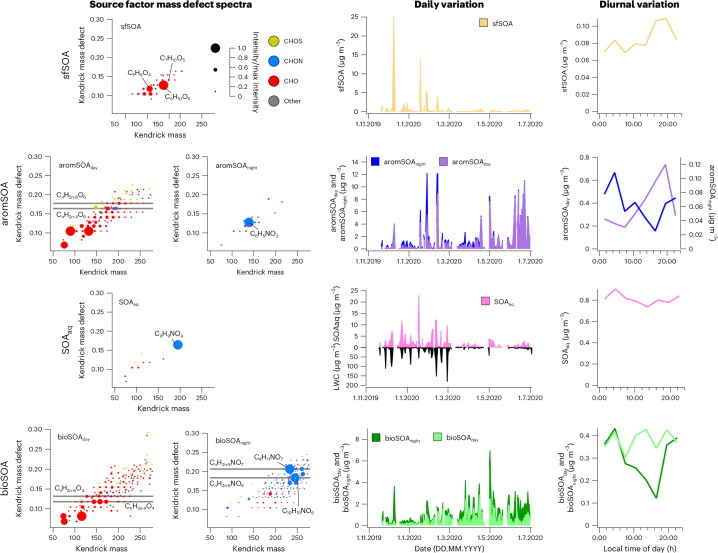
Chemical composition and temporal variability of the six secondary organic aerosol categories. Kendrick mass defect spectra show the characteristics of different sources, with near-molecular composition and relative intensity indicated by symbol colour and size, as shown in the legend (mass relative to *m*(CH_2_) = 14; only compounds with an intensity of at least 10% of maximum compound intensity). The seasonal variation is shown as daily mean concentrations and the diurnal variation as median diel cycles. Solid-fuel as well as daytime aromatic-dominated and biogenic sources are principally C_*x*_H_*y*_O_*z*_ oxidized organics. Wintertime solid-fuel SOA has prominent lignin and cellulose-like constituents, C_6_H_10_O_5_ and C_7_H_10_O_5_. Daytime aromatic-dominated SOA has products consistent with trimethylbenzene laboratory SOA (grey lines)^23^. Daytime biogenic SOA has constituents consistent with α-pinene laboratory SOA (grey lines)^14^. Nocturnal categories feature prominent nitrogen-containing species (C_*x*_H_*y*_O_*z*_N_*r*_), consistent with NO_3_ radical oxidation. Aqueous SOA is clearly enhanced in the presence of high aerosol liquid water content (LWC), linking its formation to aqueous-phase processes. Night-time biogenic SOA has products consistent with d-limonene + NO_3_ laboratory SOA (grey lines)^20^.

**Fig. 3:**
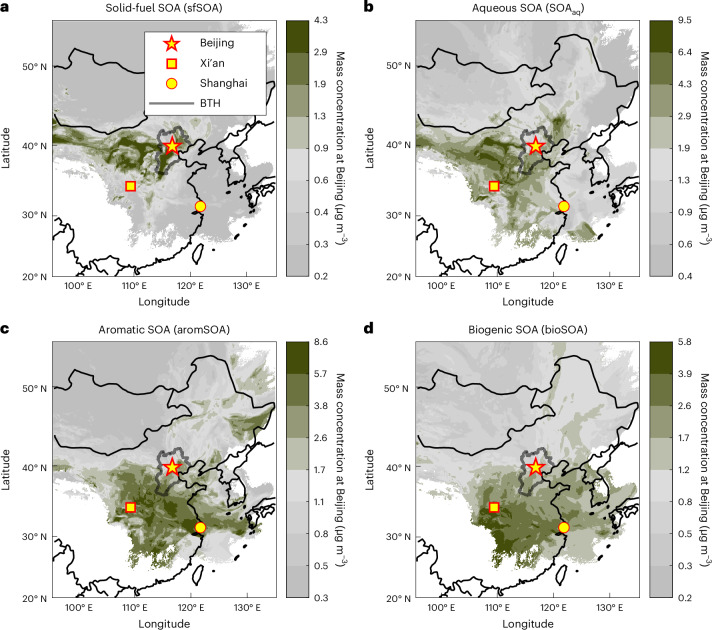
Geographical origin of SOA categories. **a**–**d**, Three-day back dispersion maps of air observed at the Beijing site (yellow star) in contact with the surface, coloured by component concentration observed at the site: solid-fuel SOA (**a**), aqueous SOA (**b**), aromatic-dominated SOA (**c**) and biogenic SOA (**d**). The average for the whole measurement period is shown. Areas contributing to above-average concentrations at the Beijing site are indicative of high emissions of the specific SOA precursors and shown in olive (below-average concentrations are shown in grey). Wintertime air masses from the Beijing–Tianjing–Hebei (BTH) region and from regions west of Beijing show high solid-fuel SOA concentrations. Aqueous SOA also associates largely with air from regions with high SO_2_ emissions, typically with very high relative humidity and thus high LWC. Summertime aromatic-dominated SOA shows regional origins from throughout the region south of Beijing, delimited roughly by a triangle defined by Beijing (yellow star), Xi’an (yellow square) and Shanghai (yellow circle). Summertime biogenic SOA also originates from the same direction but principally to the south of this region where biogenic emissions are largest.

SOA is diverse and governed by a variety of emissions and atmospheric formation processes. To identify the sources, we rely on the near-molecular composition of each SOA component, which we compare to laboratory SOA (Fig. 2 and Extended Data Figs. 6 and 7). We also rely on the temporal variability of each SOA component along with additional parameters (for example, organic marker compounds, gas-phase measurements; Fig. 2 and Extended Data Figs. 8 and 9). In Fig. 3, we determine the geographical origin of the SOA components.

In winter, solid-fuel combustion emissions contribute substantially to OA. We find that primary solid-fuel OA emissions are represented by distinct daytime and night-time components (SFOA_day_, SFOA_night_; Extended Data Fig. 6). Both SFOA_day_ and SFOA_night_ are characterized by a large contribution of C_6_H_10_O_5_—plausibly levoglucosan. Levoglucosan is emitted during solid-fuel combustion—predominantly from biomass but also from coal^37^—and during this study period the concentrations are comparable to previous years (Supplementary Fig. 2). SFOA_night_ is largely dominated by C_6_H_10_O_5_, with minor contributions of C_5_H_8_O_4_ (possibly glutaric acid). SFOA_day_ shows a higher contribution of other compounds than C_6_H_10_O_5_, such as C_7_H_10_O_5_ (found in laboratory biomass burning SOA^38^) and C_8_H_12_O_5_, as well as nitroaromatics such as C_6_H_5_NO_4_ and C_7_H_7_NO_4_. This chemical composition of SFOA_day_ is in line with aged emissions^28^. Together with the observed daytime maximum concentrations, this suggests that SFOA undergoes rapid photochemical transformation. The sum of SFOA_day_ and SFOA_night_ shows a similar temporal behaviour as solid-fuel POA (sum of biomass burning, BBOA and coal combustion, CCOA, emissions) quantified by the ToF-ACSM (Extended Data Fig. 6). Although during clean winter conditions coal combustion contributes between 65% and 96% to SFOA, more polluted episodes are strongly affected or even dominated (48–90%) by biomass burning (Extended Data Fig. 6). Additionally, an aged solid-fuel component (solid-fuel SOA, sfSOA) has substantial C_6_H_10_O_5_, but also a prominent influence of low-molecular-weight compounds (C_2–5_H_2–8_O_4_)—plausibly related to small dicarboxylic acids (Fig. 2). The solid-fuel SOA (as well as SFOA_night_ and SFOA_day_) is clearly enhanced during cold-period haze episodes and decreases substantially towards the warm season (winter mean, 15% of OA; summer mean, 2% of OA). Solid-fuel SOA and SFOA show high concentrations in air masses arriving from the Beijing–Tianjing–Hebei region, but also from the rural mountainous regions west and northeast of Beijing (Fig. 3a, Extended Data Fig. 10 and Supplementary Fig. 3), suggesting strong precursor emissions in these regions that are transported to Beijing.

Many activity sectors, including industry, energy and transportation, contribute to the emission of SOA precursors. Here we identify SOA related to emissions in the highly populated Xi’an–Shanghai–Beijing region (Fig. 3c), which show chemical characteristics indicative of aromatic precursor emissions (aromSOA; Fig. 2 and Extended Data Fig. 7). Daytime aromatic-dominated SOA (aromSOA_day_) in general shows a chemical fingerprint resembling laboratory SOA from aromatic precursors (here trimethylbenzene, TMB)^23^ reacting with OH, with prominent contributions of, for example, C_7–9_H_8–12_O_6_, C_6–7_H_8–10_O_6_ and C_6–7_H_8–10_O_5_ (Fig. 2 and Extended Data Fig. 7c). Consistent with such a formation pathway, aromSOA_day_ concentrations build up throughout the photochemically active hours of the day. Nevertheless, we do not rule out that other emissions (predominantly anthropogenic), including non-aromatic (such as alkanes from traffic, for example), contribute to this SOA category. On the other hand, night-time aromatic-dominated SOA (aromSOA_night_) is characterized by a dominant C_6_H_5_NO_3_ signal—plausibly nitrophenol peaking at night. Although aromSOA_night_ is highest during the spring, aromSOA_day_ concentrations are highest during the summer, consistent with higher irradiation and photochemical activity. In addition, the East Asian monsoon circulation probably contributes to the seasonal variability in aromatic-dominated SOA. During winter, the transport of pollution to Beijing is mainly influenced by north China, but in summer the influence extends further south throughout the Xi’an–Shanghai–Beijing region (Fig. 3c, Extended Data Fig. 10 and Supplementary Figs. 4 and 5)^39,40^. Thus, SOA from precursor sources that are mainly located in these southern regions are expected to show higher concentrations in Beijing during the summer. Air masses from regions associated with aromatic-dominated SOA have an age of up to two to three days (Supplementary Fig. 6), so long-range transport over extended time periods further facilitated by high oxidant concentrations^41–43^ enabling atmospheric processing could plausibly explain why aromSOA_day_ is strongly oxidized when arriving at the measurement site.

With increasing temperatures, biogenic-SOA precursor emissions increase, driving increased gas-phase concentrations of oxygenated organic molecules (OOM) from monoterpene and isoprene oxidation^44^. We observe an increasing biogenic-SOA concentration during the transition from winter to summer (Fig. 2). In the summer, biogenic SOA contributes an average of 27% to OA. Its concentration increases as the temperature rises (0.6 µg m^−3^ at 0 °C; 2.1 µg m^−3^ at 25–30 °C). The biogenic-SOA concentrations found here are similar to an estimate based on the concentration–temperature relation of methylbutanetricarboxylic acid (MBTCA) and pinic acid^7^, oxidation products of α-pinene (Extended Data Fig. 8). In addition, biogenic SOA correlates better than aromatic-dominated SOA with gas-phase OOM from isoprene and monoterpene oxidation (Extended Data Fig. 9). Biogenic SOA is sensitive to emissions from the forested areas in southern China (Fig. 3d and Extended Data Fig. 10), where large biogenic emission fluxes are expected^45^. Daytime biogenic SOA (bioSOA_day_) is characterized by compounds that exhibit similarities with laboratory α-pinene ozonolysis SOA such as C_8_H_12_O_4_ or C_8_H_10_O_5_ (Fig. 2 and Extended Data Fig. 7)^14^. In addition to compounds indicative of biogenic SOA from terpenes, smaller-molecular-weight compounds were also substantial contributors. These may be related to enhanced atmospheric fragmentation in the urban atmosphere or in part to other biogenic SOA precursors such as isoprene subjected to NO_*x*_ (C_2_H_4_O_3_, C_4_H_8_O_3_, C_4_H_7_NO_5_ and C_5_H_9_NO_5_)^21^. Some of the smaller-molecular-weight compounds (for example, C_2_H_4_O_3_ and C_4_H_8_O_3_) could also be fragmentation products of larger compounds from thermal decomposition in FIGAERO-CIMS during thermal desorption (Supplementary Fig. 7). Accordingly, we use the entire chemical fingerprint (including small- and large-molecular-weight compounds) to interpret the SOA sources. Although, chemically, daytime biogenic SOA shows some similarity to daytime aromatic-dominated SOA, compounds found in laboratory aromatic SOA are clearly less abundant in bioSOA_day_ than in aromSOA_day_ (Extended Data Fig. 7). During the night, biogenic SOA (bioSOA_night_) is dominated by compounds (C_8_H_11_NO_7_, C_10_H_15_NO_6_, C_10_H_17_NO_6_, C_9_H_15_NO_7_, C_10_H_15_NO_7_, C_10_H_17_NO_7_ and C_10_H_15_NO_8_) that have been identified as dominant in laboratory SOA from limonene reacting with nitrate radicals—a typical reaction pathway during the night^20^ (Fig. 2 and Extended Data Fig. 7).

SOA can also be formed by the multiphase chemistry of condensing vapours on particles, or in fog droplets. It has often been hypothesized that a substantial fraction of haze SOA in Beijing is formed in the aqueous phase^10,46–50^. However, recent estimates suggest that in Beijing during the winter, a major fraction of SOA is formed through oxidation and subsequent condensation of gas-phase precursors^51,52^. Based on our measurements, we observe an SOA type strongly associated with high particle liquid water content (LWC) concentrations (winter, 28% of OA; summer, 15% of OA), indicating that it is SOA formed in the aqueous phase (aqueous SOA, SOA_aq_; Fig. 2, *R* = 0.68). In comparison, aromatic-dominated SOA is only weakly correlated with particle LWC (${R}_{{\rm{{aromSO}{A}_{{night}}}}}$ = 0.32; ${R}_{{\rm{{aromSO}{A}_{{day}}}}}$ = 0.17) and is thus apparently not related to aqueous formation pathways. Solid-fuel SOA is enhanced in the presence of high particle LWC, although the association between solid-fuel SOA and particle LWC is quite scattered (*R* = 0.48), suggesting that other formation pathways play an important role. Air-mass backward dispersion analysis further supports the identification of aqueous SOA. Air masses with high aqueous SOA and sulfate—known to be strongly influenced by aqueous formation^4^—pass over similar regions characterized by high SO_2_ emissions (Fig. 3b and Supplementary Figs. 8 and 9). In addition, wintertime air masses with high aqueous-SOA loadings are also influenced by transport over the Bohai Sea where the air masses can take up water vapour (Extended Data Fig. 10). During the winter, aqueous SOA contributes 49% to SOA, highlighting the important role of multiphase chemistry. This is consistent with estimates that 38% of SOA during winter in Beijing is formed through condensing oxygenated organic molecules^51,52^, leaving 62% of SOA formed via other unaccounted-for formation processes, such as multiphase pathways. Aqueous SOA is dominated by C_9_H_9_NO_4_ (found in ambient cloud water^53^, possibly dimethylnitrobenzoic acid), indicating a strong influence from anthropogenic aromatic emissions. Additionally, aqueous SOA contains small-molecular-weight compounds (C_2–5_H_2–8_O_3–5_) consistent with small mono- and dicarboxylic acids, further supporting our assignment of aqueous SOA^26^.

## Sources governing OA during pollution episodes

**Fig. 4:**
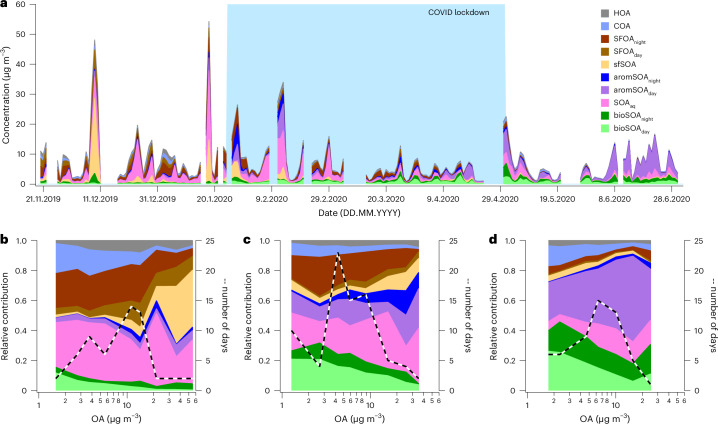
Impact of emission sources on OA air pollution. **a**–**d**, Concentration time series (**a**) and relative contribution of sources to OA at different pollution levels (**b**–**d**, daily averages). The measurement period is separated into winter (20 November 2019 to 25 January 2020, **b**), COVID lockdown (26 January 2020 to 30 April 2020, **c**), summer (1 May 2020 to 2 July 2020, **d**). The most prominent OA sources are disproportionately important during severe haze episodes, with solid fuel (especially secondary SFOA) comprising more than half of OA during wintertime haze events and aromatic-dominated SOA playing a major role during summertime haze events.

In the winter, during clean conditions, ~50% of OA consists of POA from traffic exhaust (HOA), cooking (COA), but especially SFOA, dominated by CCOA at low concentrations (Fig. 4a,b and Extended Data Fig. 6g). SOA, dominated by aqueous-phase formation processes (SOA_aq_), contributes ~50%. During pollution episodes (daily mean OA concentrations reaching >35 µg m^−3^), the contribution of SOA driven by solid-fuel SOA (38–39% of OA) increases substantially, reaching up to 80% during severe haze episodes (Fig. 4a,b). Because biomass-burning emissions dominate the primary SFOA, such emissions could also be the main driver of solid-fuel SOA during these events. Other sources contribute to winter SOA during haze episodes, including SOA related to the aqueous particle phase, aqueous SOA (17–29% of OA) and aromatic-dominated SOA (8–9% of OA). Interestingly, precursor emission sources driving increased SOA are located outside Beijing, with a substantial contribution from the Beijing–Tianjin–Hebei region (Fig. 3a and Extended Data Fig. 10).

In contrast, during clean summer conditions, OA is dominated by SOA, with POA (HOA, COA and SFOA) comprising only 19% of the OA (Fig. 4a,d). In the summer, aromatic-dominated SOA is the main driver of SOA (61%), and biogenic SOA remains a substantially smaller contributor (36%). During pollution episodes in the summer, the contribution of aromatic-dominated SOA is enhanced, contributing 38–58% to OA. Even if biogenic SOA is a relatively small contributor to OA during pollution episodes, it is clearly influenced by interactions with anthropogenic NO_*x*_ emissions. There is more bioSOA_night_ compared to bioSOA_day_ at high OA levels than at lower OA levels, indicating that during polluted conditions, biogenic SOA is preferentially produced through interactions between biogenic SOA precursors and anthropogenic NO_*x*_. This is in line with observations elsewhere showing that biogenic SOA is enhanced when interacting with urban anthropogenic emissions^54^. SOA precursors driving SOA formation are mainly emitted outside Beijing, as air masses reside over the Xi’an–Shanghai–Beijing region (Fig. 3 and Extended Data Fig. 10). Overall, SOA drives OA pollution episodes in summer and winter Beijing. However, SOA precursor emissions differ in summer and winter, with distinct sources and geographical origins outside Beijing.

## Implications

To fully understand haze in highly polluted megacities and to design targeted effective mitigation strategies, detailed molecular information is needed to identify the sources of OA, which could be particularly harmful to human health^6–8^. SOA in summer and winter is driven by chemically and geographically different precursor emission sources arriving from outside Beijing. This shows that, although our focus is on pollution within Beijing, haze is a large-scale regional phenomenon, with transport of different SOA sources over hundreds of kilometres before the particles are removed. This is in line with previous observations and model simulations^55–58^. Accordingly, to achieve substantial OA reductions, coordinated and stringent large-scale air-quality policies are required across one of the most populated regions (Xi’an–Shanghai–Beijing)^59^. Our conclusions are consistent with observations during the COVID-19 lockdown, during which reductions in traffic density, coal consumption and general economic activity did not fully mitigate pollution in Beijing driven by secondary PM_2.5_ formed in the atmosphere^60,61^. As a starting point for globally improving air quality via targeted mitigation strategies, our framework based on detailed near-molecular chemical characterization of particulate air pollution opens new research avenues for identifying aerosol sources and assessing their toxicity as well as their impact on public health.

## Methods

### Measurement site

The sampling site in Beijing is located near the west 3^rd^ Ring Road situated on the west campus of Beijing University of Chemical Technology (BUCT; 39° 56′ 31′′ N, 116° 17′ 50′′ E). The observatory is located on the top floor of a five-storey building (~20 m above ground level). The station is surrounded by residential areas with possible local emissions. Overall, the station represents a typical urban residential location in Beijing^62,63^. Between November 2019 and July 2020, a detailed chemical characterization of PM_2.5_ was performed.

### Chemical characterization of PM_2.5_

A ToF-ACSM set-up equipped with a PM_2.5_ lens and a standard vaporizer^64,65^ was used to quantitatively characterize the dry (Nafion dryer Perma Pure, MD-700-24F-3) non-refractory PM_2.5_ content and its bulk constituents (organic aerosol (OA), nitrate (NO_3_), sulfate (SO_4_), chloride (Cl) and ammonium (NH_4_)). The aerosol mass spectrometer and ToF-ACSM provides chemical fingerprints of OA that are widely used for source apportionment^3,66–68^. However, the measurement principle (vaporization at 600 °C, electron impact ionization) causes strong fragmentation of the organic compounds, leading to the detection of small fragment ions instead of molecular ions. Thus, information, in particular on SOA, is largely lost, although information on POA sources can be well retrieved. The data analysis flow is detailed in ref. ^62^. The relative ionization efficiencies (RIE) were 4.0 for NH_4_, 0.86 for SO_4_ and 1.5 for Cl (for OA a default of 1.4 was used). A composition-dependent collection efficiency (CE) was determined and used to correct the data^69^. A collocated seven-wavelength, dual-spot aethalometer (AE33, Magee Scientific Corp.) was used to measure the concentration of eBC^70^. The chemically resolved PM_2.5_ was compared to total PM_2.5_ data from the surrounding monitoring stations, and was found to be in good agreement (Supplementary Fig. 10). During the ToF-ACSM downtime, NH_4_, SO_4_, NO_3_ and Cl were gapfilled using measurements from a monitor for aerosols and gases in ambient air (MARGA, 2060R, Metrohm Process Analytics). Based on the bulk chemical composition, particle LWC values were computed using ISORROPIA^71^. In addition, PM_2.5_ values were collected on preheated quartz fibre filter samples using a HiVol sampler (24 h) between February 2018 and March 2019 (stored at −20 °C). Based on the water extracts of these filters, levoglucosan was quantified using high-performance liquid chromatography with the pulsed amperometric detector method^72,73^. MBTCA and pinic acid were quantified based on an external calibration with self-synthesized standards. Extraction was carried out with acetonitrile/water (50/50 vol/vol) in an orbital shaker for 20 min (in two steps with 250 µl and 150 µl). The combined extracts were separated by ultrahigh performance liquid chromatography (UHPLC, Vanquish Flex, Thermo Fisher Scientific) on a C18 column (Accucore 150 × 2.1 mm, 2.6-µm particle size, Thermo Fisher Scientific), and the compounds were ionized by heated electrospray ionization (operated in negative polarity) on an Orbitrap mass spectrometer (Q Exactive Focus hybrid mass spectrometer, Thermo Fisher Scientific). The eluents used were as follows: A, ultrapure water with 0.1% formic acid (vol/vol); B, acetonitrile with 0.1% formic acid (vol/vol). The gradient was set as follows: starting with 1% B for 2 min, increasing to 99% B within 13 min, holding for 2 min, decreasing to 1% B within 1 min, followed by 2 min for re-equilibration. Extracted ion chromatograms (±4 ppm) of the molecular ions ([M − H]^−^) were used for peak integration.

### Near-molecular OA characterizations using FIGAERO-CIMS

The near-molecular composition of PM_2.5_ was characterized online with FIGAERO-CIMS^13^. FIGAERO-CIMS uses soft chemical ionization, allowing for the detection of molecular ions and their chemical formulae, but not their structure. With this approach, it is able to detect a wide range of organic compounds in widely different OA types including biogenic laboratory SOA (isoprene, IEPOX, different monoterpenes and sesquiterpenes)^20,22,74,75^, laboratory anthropogenic SOA from pure components (such as catechol, trimethylbenzene, methylbenzene and toluene)^23,76^, cooking POA in indoor settings^24,77^ and ambient biomass smoke^25^, as well as complex indoor^78^ and ambient OA^26,27,79–83^. Although not all compounds in OA are detected^13^^,28,29^, compared to previously used tracer-based approaches, a much larger OA mass fraction is represented, allowing for unprecedented assessment of the main sources of SOA. Thermal evaporation can result in the fragmentation of labile organic compounds, which need then to be interpreted with caution. The FIGAERO-CIMS alternates automatically between collecting PM_2.5_ on a polytetrafluoroethylene filter (Zefon International, 25-mm diameter, 1-µm pore size) and chemically analysing the collected PM_2.5_. Post collection, the filter is moved to the desorption port, where it remains for 2 min before initiating the heating phase (dry synthetic air from a pure air generator, desorption flow = 2.3 l min^−1^). Subsequently, the filter is thermally desorbed at a heating rate of 11 °C min^−1^ for 15 min from room temperature (25–27 °C) to the maximum temperature of 190–194 °C. Afterwards, the filter is soaked at the highest set temperature for 15 min and then finally cooled to room temperature. After this first heating cycle, the filter was directly (without additional exposure) subjected to an identical second desorption cycle to measure the background signal without added particles. The vapour resulting from the desorption was ionized by the addition of iodide (generated from methyliodide subjected to an X-ray source, with an ion-molecule reaction region (IMR) pressure of 300 mbar). Finally, the ions were analysed by a long ToF mass spectrometer (LToF-MS, *m*/*z* calibration within 2 ppm, mass resolving power *m*/∆*m* – 9,000 – 11,000, Supplementary Fig. 11). Field blank measurements were performed by removing the particles from the PM_2.5_ collection stream using a high-efficiency particulate air filter.

#### Data processing

FIGAERO-CIMS data were analysed by Tofware 3.1.0. The mass spectral data [XI^−^] were corrected for fluctuations in the reagent ion ([I^−^]) as suggested by ref. ^84^:

1 $${\left[{\rm{X{I}}}^{-}\right]}_{\rm{{I}_{c}}}={\mathrm{ln}\left(1+\frac{\left[{\rm{X{I}}}^{-}\right]}{\left[{\rm{I}}^{-}\right]}\right)}$$

In the presence of a high total ion current from ions other than the reagent ion ([I^−^]), the signal of the contamination peak (C_4_H_5_ClO_3_I^−^, [ContI^−^]) appears to be suppressed (that is, a lower signal during the first desorption cycle than during the second; Supplementary Fig. 12). Thus, in the second stage, the analyte concentrations were further corrected by this peak’s signal ratio of the desorption cycles ${\left(\scriptstyle\frac{{\left[{\rm{{Cont}{I}}}^{-}\right]}_{{\rm{{I}_{c}},\,{\rm{des}}}2}}{{\left[{\rm{{Cont}{I}}}^{-}\right]}_{{\rm{{I}_{c}}},\,{\rm{des}}1}}\right)}$:

2 $${\left[{\rm{X}}^{-}\right]}_{{\rm{I+{c}_{c}}}}={\left[{\rm{X{I}}}^{-}\right]}_{{\rm{{I}_{c}}},\,{\rm{des}}1}\times \frac{{\left[{\rm{{Cont}{I}}}^{-}\right]}_{{\rm{{I}_{c}}},\,{\rm{des}}2}}{{\left[{\rm{{Cont}{I}}}^{-}\right]}_{{\rm{{I}_{c}}},\,{\rm{des}}1}}$$

Furthermore, a thermal baseline was computed for each peak for the first and second desorption cycle, which was subtracted from the respective desorption cycle:

3 $${\left[{\rm{X{I}}}^{-}\right]}_{{\rm{I+{c}_{c},\,{bs}{l}_{c}}}}={\left[{\rm{X{I}}}^{-}\right]}_{{\rm{I+{c}_{c}}}}-{\left[{\rm{X{I}}}^{-}\right]}_{{\rm{I+{c}_{c},\,{bsl}}}}$$

The thermal baseline was computed based on an algorithm presented in ref. ^85^. An example of a thermal baseline is presented in Supplementary Fig. 13.

The difference between the first and second desorption was computed:

4 $${\left[{\rm{X{I}}}^{-}\right]}_{{\rm{I+{c}_{c},\,{bs}{l}_{c},\,{bk}{g}_{c}}}}={\left[{\rm{X{I}}}^{-}\right]}_{{\rm{I+{c}_{c},\,{bs}{l}_{c},\,{des}}}1}-{\left[{\rm{X{I}}}^{-}\right]}_{{\rm{I+{c}_{c},\,{bs}{l}_{c},\,{des}2,\,{avg}}}}$$

where ${\left[{\rm{X{I}}}^{-}\right]}_{{\rm{I+{c}_{c},\,{bsl}_c,\,{des}2,\,{avg}}}}$ is the average of the second desorption before and after ${\left[{\rm{X{I}}}^{-}\right]}_{{\rm{I+{c}_{c},\,{bs}{l}_{c},\,{des}}}1}$.

Finally, ${\left[{\rm{X{I}}}^{-}\right]}_{\rm{I+{c}_{c},\,{bs}{l}_{c},\,{bk}{g}_{c}}}$ was integrated through the entire thermogram and normalized to the amount of air sampled during the preceding collection period (*V*_air_):

5 $${\left[{{\rm{X}{I}}}^{-}\right]}_{\rm{I+{c}_{c}},\,{{\rm{bsl}}}_{{\rm{c}}},\,{{\rm{bkg}}}_{{\rm{c}}}}^{\rm{s}}={\sum _{{n}_{\rm{thermo}}}{\left[{\rm{X{I}}}^{-}\right]}_{\rm{I+{c}_{c},\,{bs}{l}_{c},\,{bk}{g}_{c}}}\, {{\rm{d}}t}}\times\frac{1}{V_{\rm{air}}}$$

The field blank measurements were interpolated based on a parametrization for each peak that links the ratio of the filter measurement to the previous and following ambient measurements (‘blank fraction’) to the filter loading of the respective peak (proportional to [XI^−^] × *V*_air_) through an exponential relationship (example in Supplementary Fig. 14):

6 $${\frac{{\left[{\rm{X}{I}}^{-}\right]}_{\rm{I+{c}_{c},\,{{\rm{bsl}}}_{{\rm{c}}},\,{{\rm{bkg}}}_{{\rm{c}}}}}^{\rm{s}}\left({\rm{blank}}\right)}{{\left[{\rm{X{I}}}^{-}\right]}_{\rm{ambient}}}={y}_{0}+{A}\times \exp \left(-\frac{{\left[{\rm{X{I}}}^{-}\right]}_{\rm{ambient}}\times {V}_{\rm{air}}}{\tau }\right)}$$

with [XI^−^]_ambient_ estimated as the average of the ambient sample before and after the blank measurement. This fit was bootstrapped, leading to 100 estimates of *y*_0_, *A* and *τ*. Using these 100 combinations of the fitting parameters, 100 blank concentrations were computed for each peak [XI^−^] at each point in time, with the average used as the best estimate of the blank concentration:

7 $$\begin{array}{l}{\left[{\rm{X{I}}}^{-}\right]}_{\rm{I+{c}_{c},\,{bs}{l}_{c},\,{bk}{g}_{c},\,{bl}}}^{\rm{s}}={y}_{0}+{A}\times \exp \left(-\frac{{\left[{\rm{X{I}}}^{-}\right]}_{\rm{I+{c}_{c},\,{bs}{l}_{c},\,{bk}{g}_{c}}}\times {V}_{\rm{air}}}{\tau }\right)\\ \times \,{\left[{\rm{X{I}}}^{-}\right]}_{\rm{I+{c}_{c},\,{bs}{l}_{c},\,{bk}{g}_{c}}}^{\rm{s}}\end{array}$$

This best estimate of the field blank concentration at each time point was subsequently subtracted:

8 $${\left[{\rm{X{I}}}^{-}\right]}_{\rm{I+{c}_{c},\,{bs}{l}_{c},\,{bk}{g}_{c},\,b{l}_{c}}}^{\rm{s}}={\left[{\rm{X{I}}}^{-}\right]}_{\rm{I+{c}_{c},\,{bs}{l}_{c},\,{bk}{g}_{c}}}^{\rm{s}}-{\rm{avg}}\left({\left[{\rm{X{I}}}^{-}\right]}_{\rm{I+{c}_{c},\,{bs}{l}_{c},\,{bk}{g}_{c},\,{bl}}}^{\rm{s}}\right)$$

Previous publications suggest that estimates based on the counting uncertainty underestimate the measurement uncertainty of FIGAERO-CIMS^13,17^. Accordingly, we based the uncertainty estimates here on the repeatability measure for raw FIGAERO-CIMS thermograms (10%)^13^. After that, the uncertainty introduced by any additional computational step was estimated and propagated to the uncertainty of the raw thermograms. Peaks with poor signal-to-noise ratios were excluded from further analysis. The uncertainty of the blank was further propagated and estimated as the quadratic sum of the uncertainty related to the measurement of ${\left[{\rm{X{I}}}^{-}\right]}_{\rm{I+{c}_{c},\,{bs}{l}_{c},\,{bk}{g}_{c}}}^{\rm{s}}$ scaled with the ‘blank fraction’ and the uncertainty of the ‘blank fraction’ scaled with ${\left[{\rm{X{I}}}^{-}\right]}_{\rm{I+{c}_{c},\,{bs}{l}_{c},\,{bk}{g}_{c}}}^{\rm{s}}$.

Finally, the mass spectral matrix and the uncertainty matrix were multiplied by the molecular weight of the ion (excluding the weight of I^−^). For data mining, all peaks that were not associated to a C_*x*_H_*y*_O_*z*_N_*r*_S_*t*_ analyte iodide cluster were discarded. Finally, the signal of each organic compound (and related uncertainty) was expressed as levoglucosan-equivalent concentration by multiplication with a levoglucosan calibration factor (levoglucosan spike on a filter; Supplementary Fig. 2). We thus assume that all peaks have the same response factor as that of levoglucosan. Note that the FIGAERO-CIMS OA sources are quantified through an MLR approach relying on the ToF-ACSM OA concentrations (Supplementary Section ‘Quantification of FIGAERO-CIMS PMF factors’). Using the data analysis framework described here, the FIGAERO-CIMS OA correlates well with OA (here OA minus (HOA + COA)) measured by the ToF-ACSM (*R* = 0.87). In addition, tracers of nitrate (HNO_3_I^−^, *R* = 0.91) and sulfate (SO_3_I^−^, *R* = 0.85) also correlate well with the quantities measured by ToF-ACSM. This is in line with a previous offline filter-based study showing similarities in the temporal behaviour between FIGAERO-CIMS and ToF-ACSM^27,86^.

### Source apportionment analysis

Source apportionment was performed using the positive matrix factorization algorithm (PMF)^19^ as implemented in the multilinear engine 2 (ME-2)^87^ and controlled by the Source Finder interface (SoFi)^88,89^. PMF is a statistical unmixing model widely used in atmospheric aerosol science. In this study, OA source apportionment analyses were performed independently for the ToF-ACSM and FIGAERO-CIMS OA characterizations.

#### ToF-ACSM OA source apportionment

ToF-ACSM OA source apportionment analysis relied on the in situ observations at BUCT (1-h averages) as well as on a priori information from the literature. Previous ToF-ACSM-based studies highlight that OA in Beijing is affected by a multitude of sources^3–5,62,90^: HOA (related to traffic exhaust emissions and in general liquid-fossil-fuel combustion), COA, BBOA, CCOA, and a varying number of OOA components related to SOA. Exploratory analyses of the present dataset showed mixed sources, so we used the mass spectral signatures of specific POA sources to improve their separation (HOA, COA, BBOA and CCOA). In practice, the chemical OA fingerprints of hydrocarbon-like POA^91^, COA^91^, BBOA^4^ and CCOA^4^ were constrained as a priori information. In a preliminary PMF run with six factors covering data from 2018 to 2020, HOA and COA were constrained with an *a*-value, that is, a tolerated relative deviation from the anchor, of 0.1 and BBOA as well as CCOA with 0.2 up to *m*/*z* 85 (ramping up to an a-value of 1 at *m*/*z* 102; at higher *m*/*z* values, any value between 0 and 0.014 for BBOA and 0.016 for CCOA, respectively, was allowed) (initial guesses being the maximum divided by two). This was used to gapfill missing information on the BBOA and CCOA chemical composition at *m*/*z* > 115. In further analyses, these resulting mass spectra for BBOA and CCOA were used as constraints (a-value varied between 0 and 0.4 with an increment of 0.2) in addition to the OA chemical fingerprints for HOA and COA from ref. ^91^. (The a-value varied between 0 to 0.2 with an increment of 0.1.) We assessed the mathematical quality of the PMF solution based on the PMF residuals (res) normalized to the measurement uncertainty (*σ*)^88^. First, an overview parameter *Q*/*Q*_exp_ was computed for each solution $\left({Q}=\right.{\mathop{\sum }\nolimits_{i=1}^{m}\mathop{\sum }\nolimits_{j=1}^{n}\left(\frac{{\rm{res}}_{i,\,j}}{{\sigma }_{i,\,j}}\right)}$ and ${Q}_{\exp }={n\times m-p\times (m+n)}$, where *n* is the number of time points, *m* the number of ions, and *p* the number of factors). Although *Q*/*Q*_exp_ decreased by 32% when increasing the number of factors from 5 to 6, the decrease was clearly smaller when including a seventh factor (18%). This is in line with the more temporal structure in the measurement error-weighted PMF residuals for five than six or seven factors (Supplementary Fig. 15). Although the six-factor solution resolved two OOA components with different chemical fingerprints, these factors were mathematically further split when including a seventh factor. Because the results were not improved by including a seventh factor, here we present a six-factor solution (HOA, COA, BBOA, CCOA and two free factors). For the final source apportionment analysis, 28 day chunks of data (for each 81 PMF runs with randomly chosen a-values, see above) were used at a time (shifted by two days) until the entire study period was covered (rolling PMF^32,89,92–94^). The free/unconstrained factors were sorted based on their fractional content of *m*/*z* 44 (f44). For each window, the correlation coefficient (*R*_pearson_) between eBC and the combustion-related factors was computed as eBC = *a* × HOA + *b* × CCOA + *c* × BBOA. The lower threshold for solution acceptance was based on three median absolute deviations from the median *R*_pearson_. For periods during which the FIGAERO-CIMS was operating but not the ToF-ACSM, we interpolated the approximate HOA concentrations based on the eBC concentration (HOA_approx_ = 0.37 × eBC), and COA was approximated based on a parametrization of the COA/HOA ratio $\left({\rm{COA}}_{\rm{approx}}\right.={({\rm{base}}+\frac{(\max -{\rm{base}})}{1+{(\frac{{{\rm{xhalf}}}}{{\rm{{HOA}}_{\rm{approx}}}})}^{\rm{rate}}})\times {\rm{HOA}}_{\rm{approx}}}$; base = 174.9, max = 1.1, rate = 0.9, xhalf = 0.0023; Supplementary Fig. 16). In a last step, we assumed, based on ref. ^95^, that HOA and COA have a response factor (RIE × CE) between two and three times larger than the default RIE of OA (RIE_OA, default_ = 1.4, CE = 0.5) leading to an RIE × CE of 1.5–2 (RIE of HOA and COA of 3.5). The chemical fingerprints and time series of the resolved OA components, as well as their markers, are presented in Supplementary Figs. 17–19 (scatterplots between selected OA components and their markers are displayed in Supplementary Fig. 20). Both ToF-ACSM OOA components correlate with all FIGAERO-CIMS SOA factors (Supplementary Fig. 21). However, one OOA component correlates better with FIGAERO-CIMS sfSOA, aromSOA_night_ and SOA_aq_, whereas the other correlates better with bioSOA_night_.

#### Particle-phase FIGAERO-CIMS OA source apportionment

Given the lack of well-understood near-molecular source compositions, the FIGAERO-CIMS source apportionment analysis did not rely on a priori information. *Q*/*Q*_exp_ is reduced when increasing the number of factors and the selected eight-factor solution has a *Q*/*Q*_exp_ of 0.9. When introducing more than nine factors, *Q*/*Q*_exp_ does not decrease substantially more (<5%). With more detailed assessments based on the change in the measurement uncertainty-weighted PMF residuals, we found that the PMF explains the data increasingly better when increasing the number of factors up to eight, but there is no further substantial improvement beyond nine factors (Supplementary Fig. 22). We thus examined PMF solutions with up to nine factors, presented here though an eight-factor solution given its best environmental interpretability. We performed a sensitivity analysis based on 200 bootstrapping PMF runs. The factors were identified based on their time-series correlation (*R*_pearson_) with a base case. The lower threshold for solution acceptance was based on three median absolute deviations from the median *R*_pearson_. The chemical compositions of the factors are presented in Fig. 2 and Extended Data Figs. 6 and 7, and the temporal variation in Fig. 2, Extended Data Fig. 6 and Supplementary Fig. 23.

#### Quantification of FIGAERO-CIMS PMF factors

Because the sensitivity of FIGAERO-CIMS depends on the chemical composition of a molecule, the response factors for the identified FIGAERO-CIMS factors cannot be assumed to be the same. We aimed to determine a response factor for each FIGAERO-CIMS factor for improved quantification. To that end, we performed MLR relating the FIGAERO-CIMS factor time series to the ToF-ACSM data (equation (9)). Plausibly, the FIGAERO-CIMS cannot efficiently measure the hydrocarbon components that constitute the major parts of traffic and cooking emissions. Yet, it has been shown that other constituents of cooking emissions can be detected by FIGAERO-CIMS^24^. In the present dataset we did not identify an HOA or COA factor based on the FIGAERO-CIMS data; for example, no FIGAERO-CIMS factor shows a lunch- and dinner-time peak characteristic of COA (ToF-ACSM COA; Supplementary Fig. 19). On the other hand, HOA and COA can be well quantified by the ToF-ACSM data (section ‘ToF-ACSM OA source apportionment’). We thus subtracted HOA and COA (estimated via ToF-ACSM OA analyses) from OA and used this difference (OA(*t*) minus (HOA(*t*) − COA(*t*))) as the reference concentration (µg m^−3^) for the MLR to quantify the FIGAERO-CIMS factors (absolute signal), instead of using the entire ToF-ACSM OA:

9 $$\begin{array}{l}{\rm{OA}}\left(t\right)-({\rm{HOA}}\left(t\right)+{\rm{COA}}\left(t\right))\\={\rm{normal}}\left(\sum _{k}\frac{{\rm{{OA}}}_{k}^{\rm{FIGAERO}}\left(t\right)}{{R}_{k}^{\rm{FIGAERO}}},\,\left({\rm{OA}}(t)-{\rm{HOA}}(t)-{\rm{COA}}(t)\right)\times {\rm{rel}}_{\rm{err}}\right)\end{array}$$

${\rm{OA}}_{k}^{\rm{FIGAERO}}\left(t\right)$ represents the time series of FIGAERO-CIMS OA factors, ${R}_{k}^{\rm{FIGAERO}}$ their response factors, and rel_err_ the approximate uncertainty of OA minus (HOA + COA), which is assumed to be 10%. MLR was performed with the advanced statistical software STAN^96^ via Hamiltonian Markov chain Monte Carlo sampling from the posterior distribution of our model given the data (equation (9)). Thereby, *R*_*k*_ combinations were drawn in proportion to their posterior probability. Their variability thus provides direct uncertainty estimates of *R*_*k*_. In a preliminary analysis, the sum of SFOA_day_ and SFOA_night_ from the FIGAERO-CIMS (FIGAERO-SFOA) correlates well with the sum of BBOA and CCOA from the ToF-ACSM (ToF-ACSM-SFOA). We thus assume that FIGAERO-SFOA is the same as ToF-ACSM-SFOA. However, the preliminary quantification approach overall results in higher FIGAERO-SFOA concentrations compared to the ToF-ACSM-SFOA (${\rm{SFOA}}_{\rm{day}}^{\rm{FIGAERO}}+{\rm{SFOA}}_{\rm{night}}^{\rm{FIGAERO}}$ = 1.6 × [BBOA + CCOA]). During the summer, SFOA from FIGAERO-CIMS is considerably lower than BBOA + CCOA from ToF-ACSM. During this period, BBOA dominates the sum of BBOA and CCOA. Plausibly, summertime BBOA is mixed with other OA components because in previous studies BBOA could not be identified during summer^62^. We thus optimized SFOA^FIGAERO^ such that FIGAERO-SFOA was similar to the ToF-ACSM-SFOA concentration for November 2019 to March 2020 (with an assumed relative error of 5%—rel_err2_; equation (10)):

10 $$\begin{array}{l}{\rm{mean}}_{\rm{{Nov}-{Mar}}}\left({\rm{BBOA}}\left(t\right)+{\rm{CCOA}}\left(t\right)\right)\\={\rm{normal}}\left({\rm{mean}}_{{\rm{{Nov}-{Mar}}}}\left(\frac{{\rm{SFOA}}_{\rm{day}}^{\rm{FIGAERO}}\left(t\right)}{{R}_{{\rm{SFOA}}_{\rm{day}}}^{\rm{FIGAERO}}}+\frac{{\rm{SFOA}}_{\rm{night}}^{\rm{FIGAERO}}\left(t\right)}{{R}_{{\rm{SFOA}}_{\rm{night}}}^{\rm{FIGAERO}}}\right),\,\sigma \right),\\\,\sigma ={\rm{mean}}_{{\rm{{Nov}-{Mar}}}}\left({\rm{BBOA}}\left(t\right)+{\rm{CCOA}}\left(t\right)\right)\times {\rm{rel}}_{{\rm{err}}2}\end{array}$$

In Supplementary Fig. 24 we compare the response factors of the FIGAERO-CIMS OA components to a bulk OA response factor (*R*_bulk_) assuming that all components have the same response factor (via linear regression between the sum of all FIGAERO-CIMS OA components and OA from the ToF-ACSM). OA minus (HOA + COA) modelled by the FIGAERO factors, corrected with their respective response factors, reconstructs OA minus (HOA + COA) measured by the ToF-ACSM (slope = 0.87, *R* = 0.87; Supplementary Fig. 25), and the MLR residuals do not depend on the (HOA + COA) fraction of OA. With that approach we found the FIGAERO-CIMS response factors to the eight OA sources vary with a factor of ~10 (Supplementary Fig. 24). We note that this is an approach to estimate the concentrations of the different OA sources/factors, not of the single molecules therein. The range of estimated bioSOA concentrations is similar to estimates from a tracer-based approach using MBTCA and pinic acid (oxidation products of terpenes) from offline filter analyses from 2018/2019 (assuming a bioSOA/[MBTCA + pinic acid] ratio of 0.126 µg bioSOA/ng [MBTCA + pinic acid]^7^ (Extended Data Fig. 8).

We performed a Monte Carlo sensitivity assessment on the RIEs of HOA, COA and SFOA (BBOA, CCOA), as well as the relative response factors of the FIGAERO OA factors, with a total of 3,600 runs (Supplementary Fig. 24). We designed three RIE scenarios and accounted for the uncertainties of both the respective RIEs (POAs) and response factors (all FIGAERO OA factors; Supplementary Fig. 24):

1. Default RIEs for POA: RIE_HOA_ = 1.4 ± 0.3, RIE_COA_ = 1.4 ± 0.3, RIE_SFOA_ = 1.4 ± 0.3
2. Adapted RIEs for HOA and COA: RIE_HOA_ = 3.5 ± 0.5, RIE _COA_ = 3.5 ± 0.5, RIE_SFOA_ = 1.4 (ref. ^95^)
3. Adapted RIEs for POA: RIE_HOA_ = 5.74 ± 1.95, RIE_COA_ = 4.55 ± 1.55, RIE_SFOA_ = (4.44 ± 1.51 or 5.55 ± 1.89) (ref. ^97^).

**Extended Data Fig. 4:**
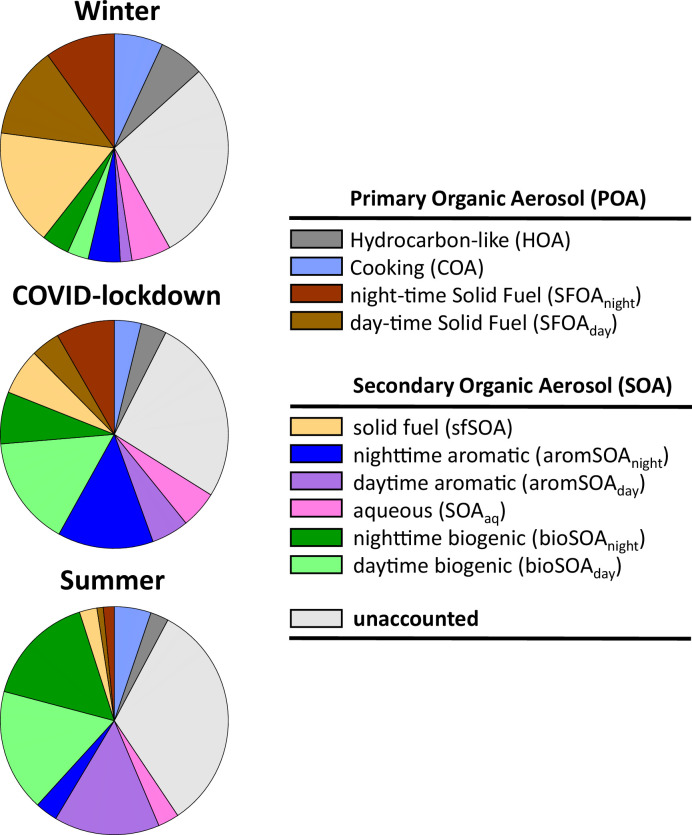
Relative contribution of POA and SOA types to OA via direct quantification of the FIGAERO-CIMS. The concentrations of FIGAERO-CIMS OA types are determined via the direct quantification approach (see methods and Extended Data Fig. 5) and combined with Hydrocarbon-like OA (HOA) and Cooking OA (COA) from the ToF-ACSM. This approach leaves the OA mass fraction that is neither quantified by the ToF-ACSM (HOA + COA) nor directly measured by the FIGAERO-CIMS as unaccounted (winter 29%, COVID lockdown 26%, summer 33%).

**Extended Data Fig. 5:**
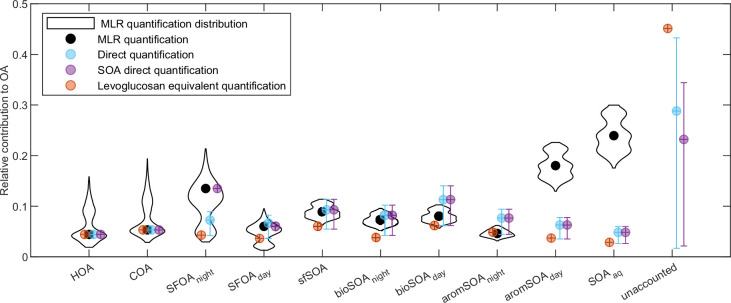
Sensitivity assessment of relative contribution of POA and SOA types to OA during the measurement period. Results from the sensitivity assessment also accounting for uncertainties in the FIGAERO-CIMS response factors are shown as violin plots. Values based on the MLR quantification setting used in the main text are shown as a black dot (violin plots show the range from 1^th^ to 99^th^ percentile from a Monte Carlo simulation with three scenarios totalling 3600 runs). Results reported as levoglucosan equivalent concentrations are reported as orange dots. In addition to the levoglucosan-equivalent quantification, the direct quantification approach combines our own levoglucosan calibration with response factors relative to levoglucosan. The results from direct quantification of all FIGAERO-CIMS factors are displayed in blue and of all FIGAERO-CIMS SOA factors in violet (the uncertainty displayed is based on 1 standard deviation of response factors relative to C_6_H_10_O_5_ in the data presented by Ye et al.^28^, parametrized based on a moving average across the mass spectrum using 40 ions at a time). With our MLR-based approach combining FIGAERO-CIMS and ToF-ACSM, we quantify which SOA types are behind the OA mass not accounted for by the directly quantified FIGAERO-CIMS OA mass.

**Extended Data Fig. 6:**
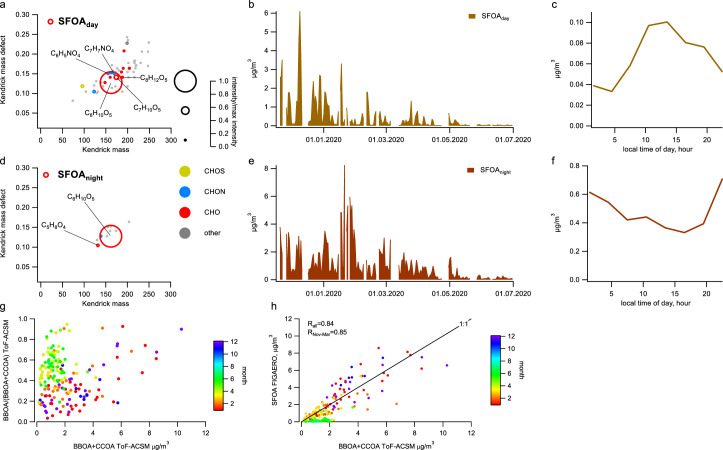
Characteristics of solid-fuel POA and the impact of biomass burning and coal combustion. **a**–**h**, Comparison between solid-fuel POA (SFOA_day_+SFOA_night_) (FIGAERO-CIMS OA data) to the sum of biomass burning and coal combustion POA resolved (ToF-ACSM) (daily means). During winter CCOA dominates SFOA at low and BBOA dominates at high SFOA (or BBOA+CCOA) concentrations. During the warm season, the ToF-ACSM based estimate (BBOA+CCOA, related to mostly BBOA) is larger than the FIGAERO-CIMS based estimated (SFOA). The chemical composition is displayed as Kendrick mass defect plots of the neutral compound with CH_2_ base. Compounds that are at least 10% of the maximum compound intensity are size-coded with the compound’s intensity normalized to the highest intensity compound and are color-coded with compound families: C_x_H_y_O_z_: red, C_x_H_y_O_z_N_r_: blue, C_x_H_y_O_z_S_r_: yellow, other: grey. Compounds with an intensity between 5 and 10% of the maximum compound intensity are shown as small unisized solid grey dots (others are not displayed). The seasonal variation is shown as daily mean concentrations and the diurnal variation as median diel cycles.

**Extended Data Fig. 7:**
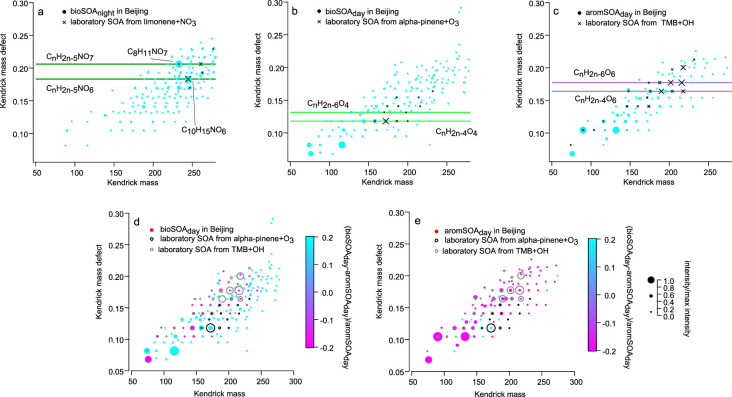
Chemical composition of biogenic-SOA and daytime aromatic-dominated-SOA. **a**–**e**, The chemical composition of biogenic-SOA (bioSOA_day_, bioSOA_night_) and daytime aromatic-dominated-SOA (aromSOA_day_) is displayed as Kendrick mass defect plots of the neutral compound with CH_2_ base. For comparison the composition of laboratory SOA of trimethylbeneze (TMB) - (reacted with OH), α-pinene (reacted with ozone), and limonene (reacted with NO_3_ radicals) is displayed. Top panel: Symbols are size-coded with a compounds intensity normalized to the highest intensity compound, compounds with intensity < 10% of maximum compound intensity are discarded, the symbols are color-coded with compound families). The violet line refers to common trimethylbenze SOA compounds from reaction with OH, the dark green line refers to common SOA compounds from limonene reacted with NO_3_ radicals, and the light green line refers to common α-pinene ozonolysis SOA compounds. Bottom panel: Symbols are size-coded with a compounds intensity normalized to the highest intensity compound and color-coded with relative difference in the normalized intensity between bioSOA_day_ and aromSOA_day_ (compounds with intensity < 10% of maximum compound intensity are discarded). This comparison illustrates that 1) bioSOA_night_ has a distinct chemical composition similar to limonene reacted with NO_3_ radicals (an important reaction pathway during the night), 2) different compounds are characteristic for bioSOA_day_ and aromSOA_day_ and that 3) many of the peaks characteristic for bioSOA_day_ are found to be important for laboratory α-pinene – biogenic - SOA and similarly for aromSOAday and TMB – anthropogenic - SOA.

**Extended Data Fig. 8:**
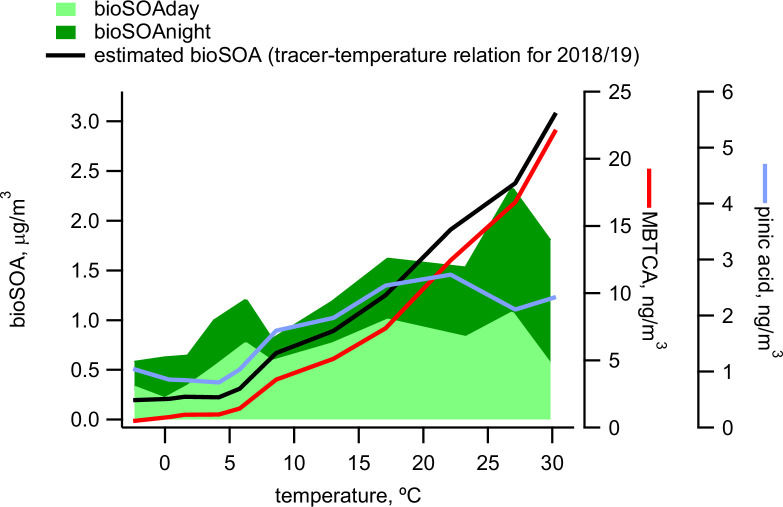
Biogenic-SOA and concentrations at different temperatures. bioSOAday and bioSOAnight are displayed as stacked green shaded areas. They are compared to biogenic-SOA concentrations at different temperatures estimated using offline filter analysis of MBTCA and pinic acid for 2018/19 (assuming a bioSOA/(MBTCA+pinic acid) ratio of 0.126 µg bioSOA per ng MBTCA+pinic acid^7^).

**Extended Data Fig. 9:**
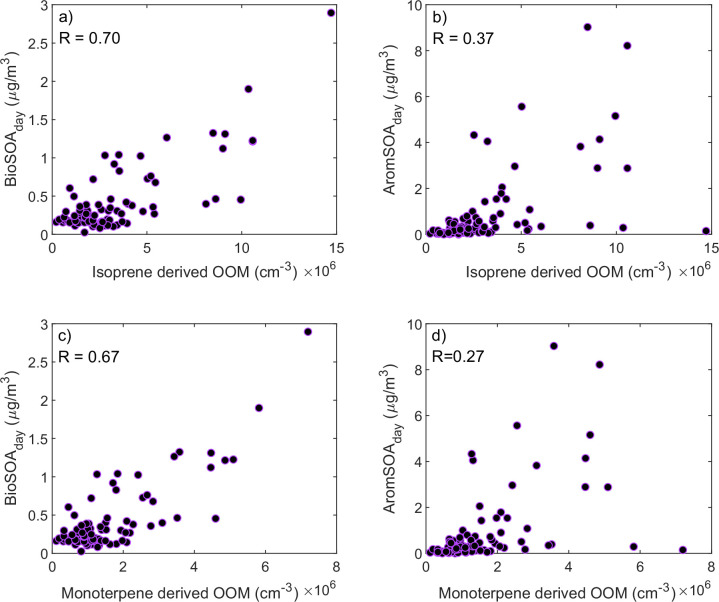
Relation between biogenic and aromatic-dominated-SOA to gas-phase Oxygenated Organic Molecules from the oxidation of isoprene and monoterpene. **a**–**d**, Biogenic-SOA correlates better than aromatic-dominated-SOA with gas-phase Oxygenated Organic Molecules (OOM) from isoprene (**a**,**b**) and monoterpene (**c**,**d**) oxidation. Correlation coefficients shown in the figure. Here the period 20.11.2019-27.04.2020 is displayed during which the daily mean temperature did not exceed 20 °C. Up to 20 °C pinic acid (more volatile) and MBTCA (less volatile) - molecules widely used as bioSOA markers – correlate well but at higher temperatures the correlation deteriorates, likely because of gas-to-particle partitioning (Supplementary Fig. 26). Such effects also impact the gas-phase to particle-phase relation and thus only days with a mean temperature lower than 20 °C are used for this comparison.

**Extended Data Fig. 10:**
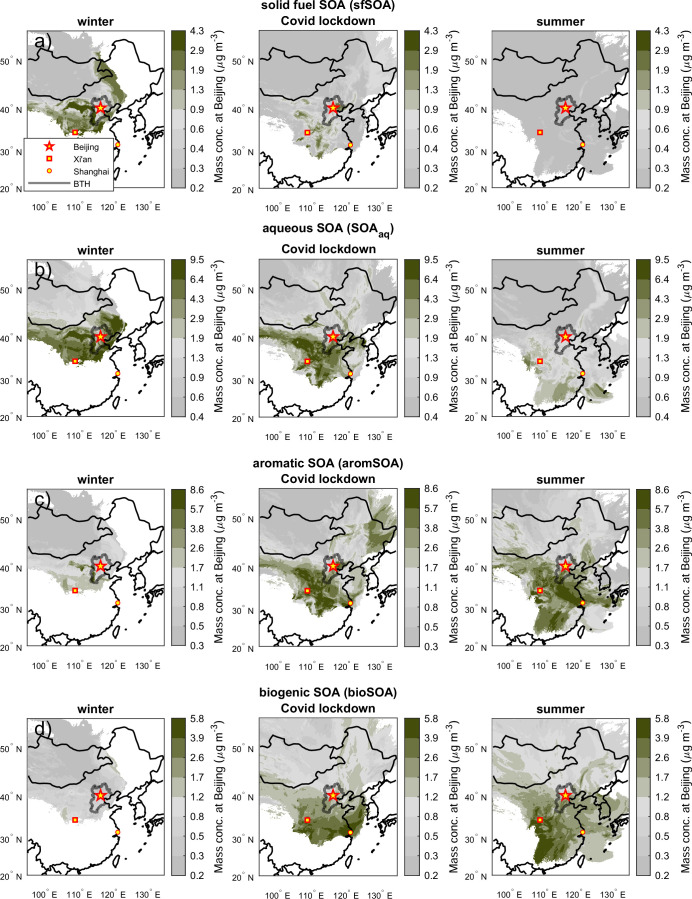
Seasonal geographical origin of secondary organic aerosol categories. 3-day back dispersion maps of air observed at the Beijing site (yellow star) in contact with the surface, colored by component concentration observed at the site. Areas contributing to above-average concentrations at the Beijing site are indicative of high emissions of the specific SOA precursors and shown in olive (below-average concentrations are shown in grey).

The results illustrate that the relative contribution of the different SOA components is subject to uncertainty, but that this does not affect our findings and conclusions (Extended Data Figs. 4 and 5).

In addition, we compared the MLR-based quantified FIGAERO-CIMS OA factors (equations (9) and (10)) to (1) levoglucosan-equivalent concentrations assuming the same response factor for all compounds and (2) a direct quantification of the FIGAERO-CIMS OA factors (Extended Data Figs. 4 and 5). For the latter direct quantification approach, we relied on our own levoglucosan calibration together with a parametrization of the response factors relative to levoglucosan (rRF) as a function of *m*/*z*, ${({\rm{rRF}}(m/z)=a+b\times (\exp (-{(\frac{m/z-c}{d})}^{2})))}$, based on data from the literature^28^ (central estimate: *a* = 0.3, *b* = 0.66, *c* = 249.17, *d* = 87.95; upper limit: *a* = 0.33, *b* = 0. 86, *c* = 235.10, *d* = 115.37; lower limit: *a* = 0.23, *b* = 0.54, *c* = 259.89, *d* = 63.77).

### Characterization of gas-phase OOM

The gas-phase oxygenated organic molecules (OOM) were characterized by a nitrate-based CIMS (NO_3_-CIMS, here equipped with an LTOF mass spectrometer with a mass resolution of 8,000–12,000)^98,99^. The configuration and calibration of this instrument have been described previously^100,101^. In brief, the NO_3_-CIMS was calibrated with a known amount of sulfuric acid^102^ and the OOM assumed to have the same response because their structures are unknown and thus cannot be calibrated for. The OOM concentrations were then computed by normalization to the reagent ions and subsequent scaling with the response factor.

The OOM related to isoprene oxidation and monoterpene oxidation were identified based on a decision tree designed using atmospheric and laboratory experiments^44,51^. Essentially, the isoprene oxidation products were identified based on a list of compounds presented in the literature^44,51^, and the monoterpene oxidation products were identified as compounds with a carbon number of 10, an equivalent oxygen number of at least 4, and a double bond equivalent between 2 and 4.

### Spatial distribution of emissions

We used a concentration-weighted trajectory (CWT) method to study the spatial distribution of the precursor sources for the different factors. Instead of trajectories, we used potential emission sensitivity (PES) fields, which were calculated using a Lagrangian particle dispersion model FLEXPART version 9.02 (ref. ^103^) with European Centre for Medium-Range Weather Forecasts (ECMWF) operational forecast data (0.15° horizontal resolution, 137 vertical levels and 1-h temporal resolution) as the meteorological input. In the FLEXPART model simulations, 50,000 tracer particles were initially distributed evenly between 0 and 100 m above the measurement site and then followed backwards in time for 72 h. The output PES fields (domain: 20–60° N 95–135° E; horizontal resolution, 0.05°) contain the residence times of the air mass (tracer particles) above the simulation grid cells. In the CWT method, we assigned each grid cell with a concentration value (*C*_*ij*_), which represents the expected concentration at the measurement site if an air mass passes over said grid cell upon its arrival to the station. The *C*_*ij*_ value was calculated based on the observed concentrations (*C*_*t*_) and the air mass residence times (*τ*):

11 $${C}_{{ij}}=\frac{\sum _{t}{C}_{t}{\tau }_{{tij}}}{\sum _{t}{\tau }_{{tij}}}$$

where *C*_*t*_ is the observed concentration at time *t*, and *τ*_*tij*_ is the residence time of the air mass over the *ij*th grid cell obtained from the PES field for a tracer release at time *t*. Because the precursor emissions for the different factors are ground-based, we used PES fields that only include the residence times of the tracer particles residing within 500 m above ground level. In addition, *C*_*ij*_ values are only shown for grid cells containing data from more than ten different observation times.

## Online content

Any methods, additional references, Nature Portfolio reporting summaries, source data, extended data, supplementary information, acknowledgements, peer review information; details of author contributions and competing interests; and statements of data and code availability are available at 10.1038/s41561-024-01493-3.

### Supplementary information

Supplementary Information Supplementary Figs. 1–26.

## Data Availability

The full dataset used in the figures is publicly available at 10.5281/zenodo.10977390 (ref. ^104^).

## Code availability

Source Finder (SoFi) is available via its distributor Datalystica Ltd (www.datalystica.com). Tofware is available via its distributors Tofwerk AG and Aerodyne Inc. FLEXPART is available via flexpart.eu. Mapping Tools for MATLAB, used for plotting the maps for the positioning data, are available from MathWorks File Exchange (https://www.mathworks.com/).
