# Supplementary material for: Substantial contribution of transported emissions to organic aerosol in Beijing
Source: Nat Geosci. 2024 Aug 8;17(8):747–54. doi: 10.1038/s41561-024-01493-3 (PMC11315673; doi:10.1038/s41561-024-01493-3)
Supplement: Supplementary file 1 — Supplementary Figs. 1–26. [file 41561_2024_1493_MOESM1_ESM.pdf]

nature geoscience

Supplementary information https://doi.org/10.1038/s41561-024-01493-3

Substantial contribution of transported emissions to organic aerosol in Beijing

In the format provided by the authors and unedited

SUPPLEMENTARY INFORMATION FOR

SUBSTANTIAL CONTRIBUTION OF TRANSPORTED EMISSIONS TO ORGANIC AEROSOL IN BEIJING

Kaspar R. Daellenbach et al.

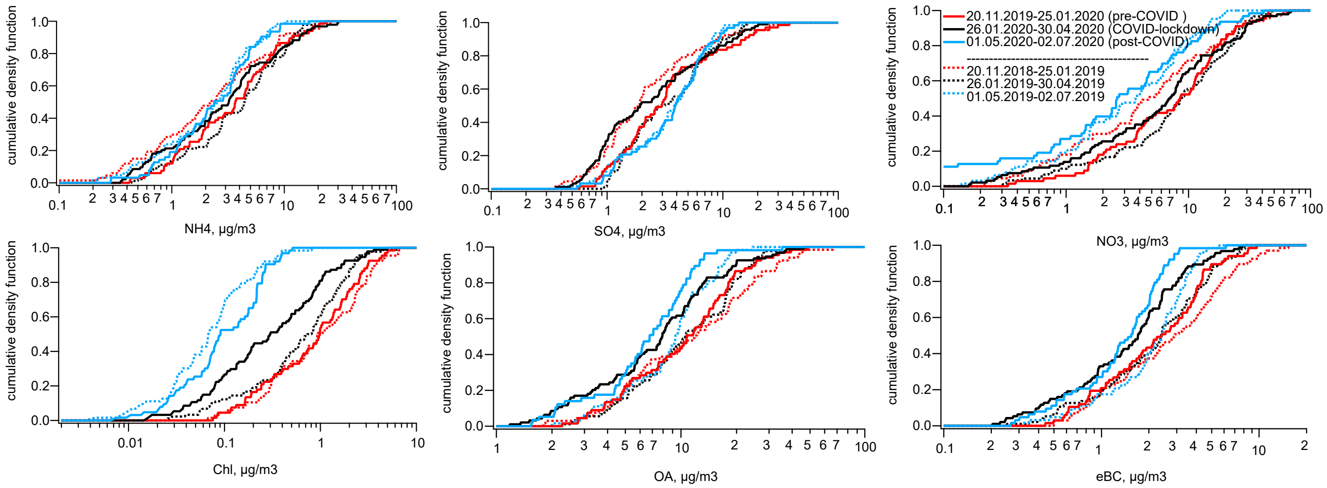

Figure SI 1: Impact of COVID-lockdown on concentrations of PM2.5 constituents. Same periods are compared between 2018/2019 and 2019/2020.

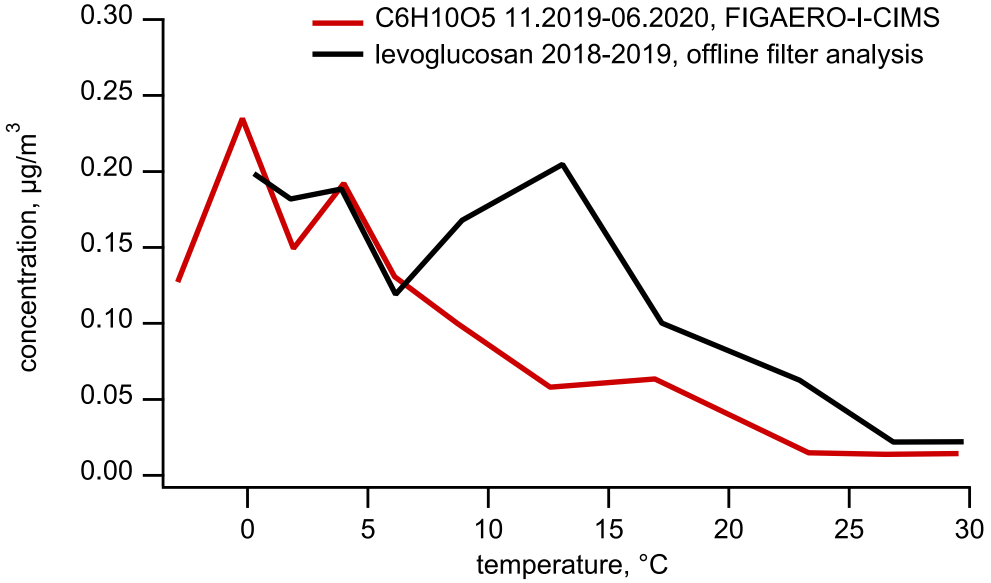

Figure SI 2: Levoglucosan concentration - atmospheric temperature relation: For 02.2018 – 03.2019 levoglucosan concentrations were determined based on offline filter analysis using high-performance liquid chromatography with the pulsed amperometric detector method. For 11.2019 – 06.2020 instead the quantified $C_6H_{10}O_5$ signal (detected as $C_6H_{10}O_5I^-$) from in-situ FIGAERO-CIMS is used (quantified using a calibration series of levoglucosan).

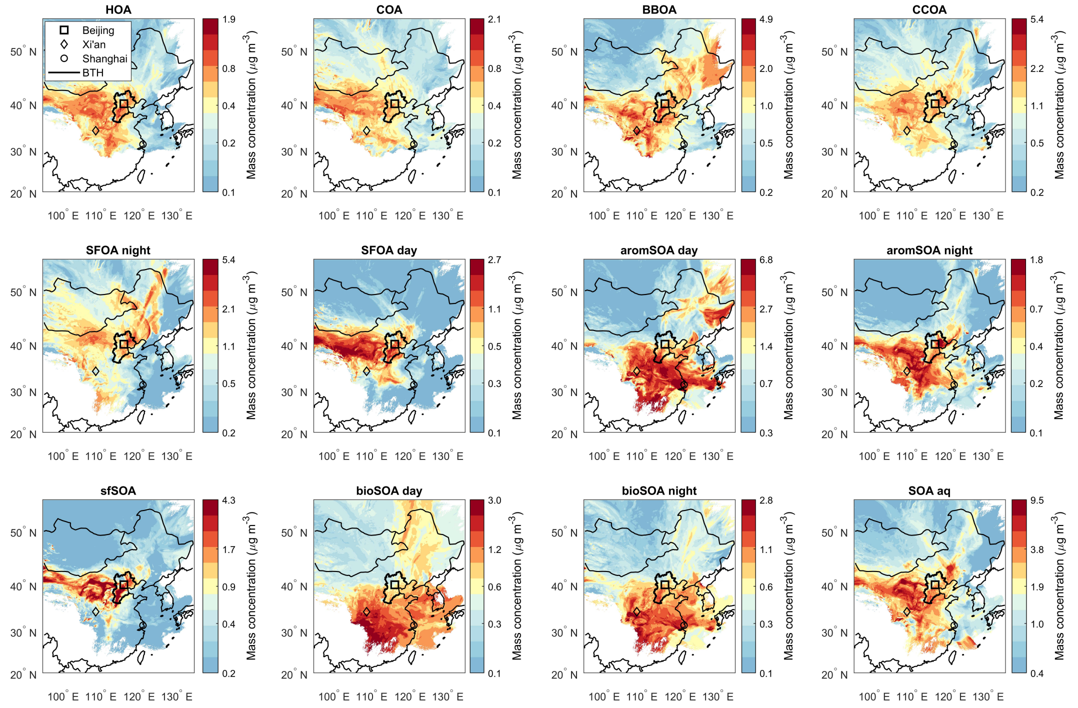

Figure SI 3: 3-day backward dispersion maps of air observed at the Beijing site in contact with the surface, colored by OA component concentration observed at the site. The average for the whole measurement period is shown. High values over specific areas suggest strong precursor emissions in these areas (or within the typical transport route passing over this area). The middle of the colorbar shows the mean value of the component over the whole observation period and the axis extends to 5 times the mean and 0.2 times the mean in logarithmic scale. Overall lighter colors and less pronounced directional preference are indicative of increasing influence from local emissions (e.g. COA) as the concentrations of locally emitted compounds do not strongly depend on the previous movements of the air mass.

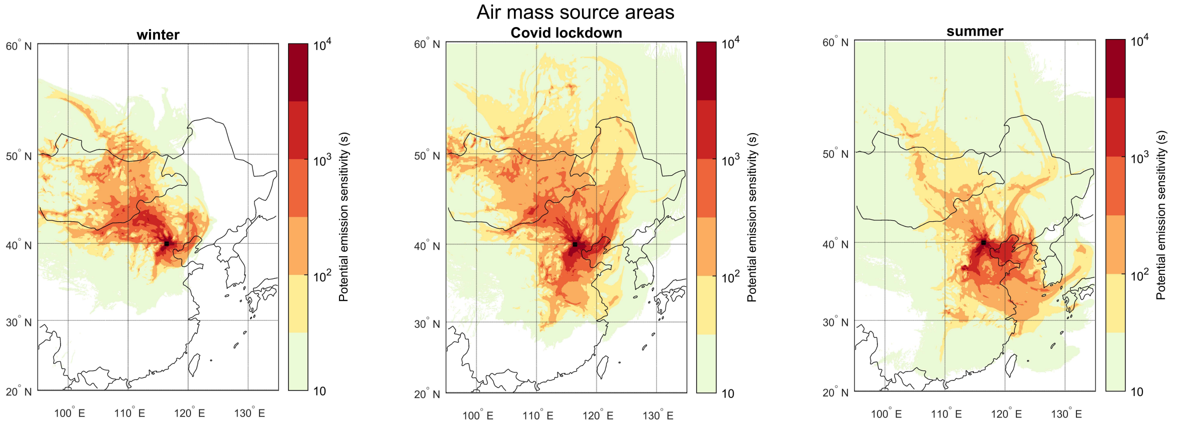

Figure SI 4: Average potential emission sensitivity fields displaying the typical residence times of air masses over different regions prior to their arrival at Beijing during the three different time periods. A clear transition from predominantly northwesterly and local air masses in winter to a more southeasterly influence during summer is observed due to the East Asian monsoon cycle.

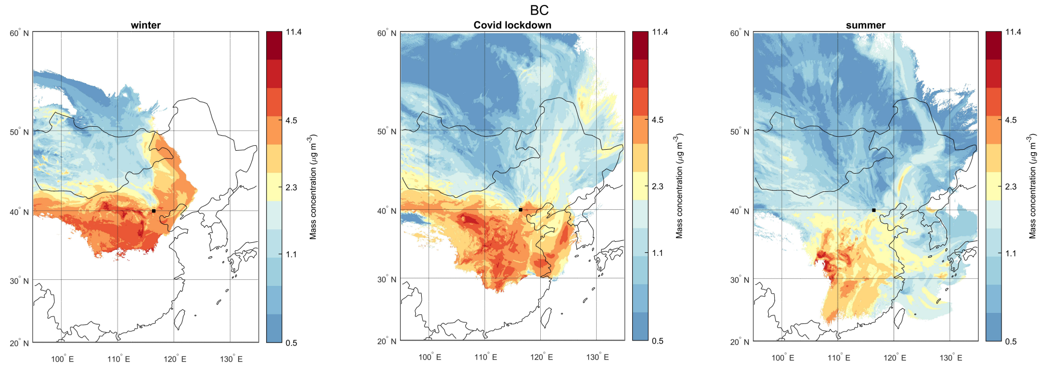

Figure SI 5: 3-day backward dispersion maps of air observed at the Beijing site in contact with the surface, colored by eBC – used as a pollution transport marker - concentration observed at the site. The average for the three different time periods is shown. High values over specific areas suggest strong emissions in these areas (or within the typical transport route passing over this area). The center of the colorbar shows the mean value of sulfate over the whole observation period and the axis extends to 5 times the mean and 0.2 times the mean in logarithmic scale.

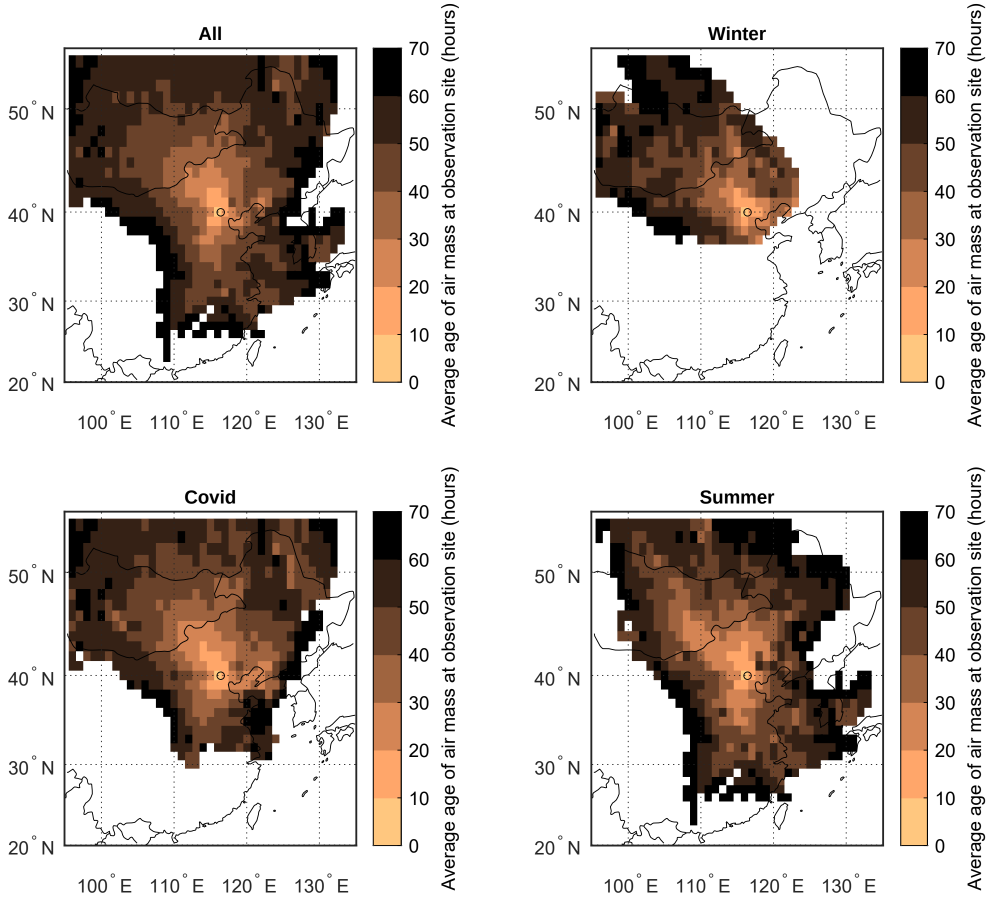

Figure SI 6: Average age of air mass arriving to Beijing for different time periods: Average time air masses take to arrive to the receptor site in Beijing after leaving a specific grid cell.

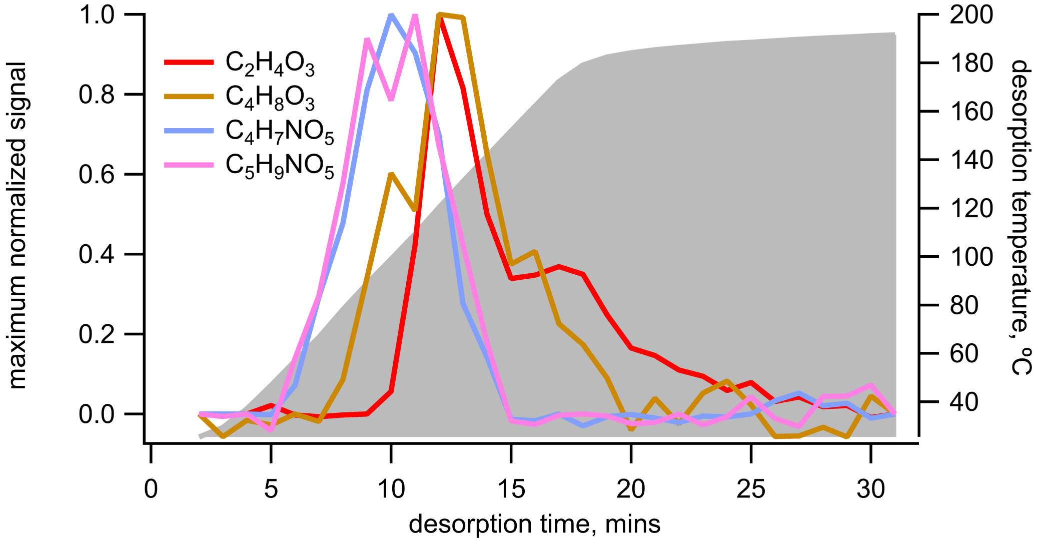

Figure SI 7: FIGAERO-CIMS thermograms of selected compounds on 11.06.2020 00:20.

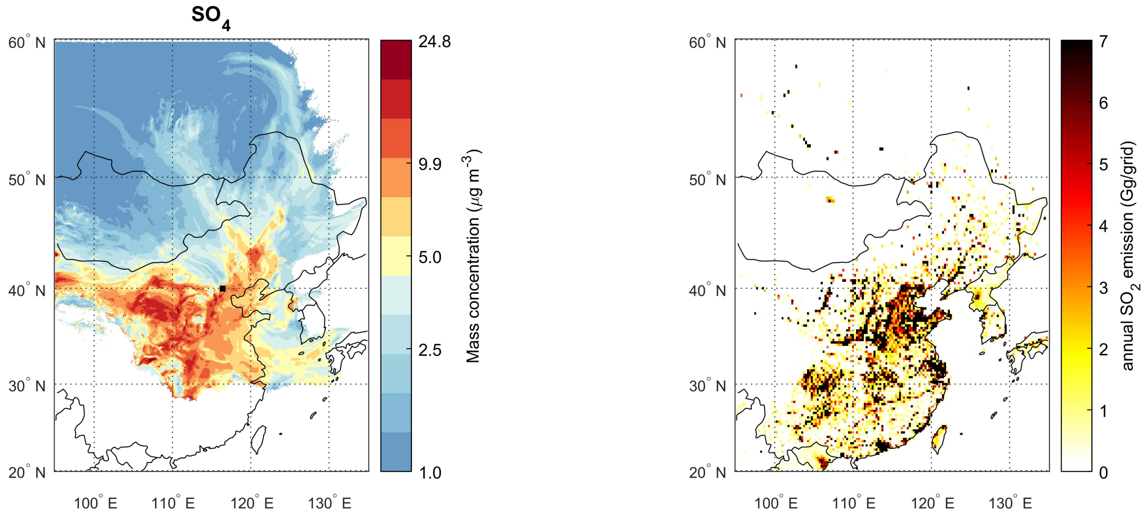

Figure SI 8: 3-day backward dispersion maps of air observed at the Beijing site in contact with the surface, colored by sulphate concentration observed at the site in comparison to the SO_2 emissions. The average for the whole measurement period is shown. High values over specific areas suggest strong precursor emissions in these areas (or within the typical transport route passing over this area). The middle of the colorbar shows the mean value of sulfate over the whole observation period and the axis extends to 5 times the mean and 0.2 times the mean in logarithmic scale (left panel). Annual emissions of SO_2 (precursor of sulfate) for the year 2010 from the MIX emission inventory [1]. Over most regions, the estimated source areas of the emissions match well with the emission inventory, suggesting robust performance of the approach. However, contributions from some regions e.g. those south of the Mongolian border in the west, are likely overestimated due to air masses arriving from these regions consistently passing over high emission closer to the measurement station.

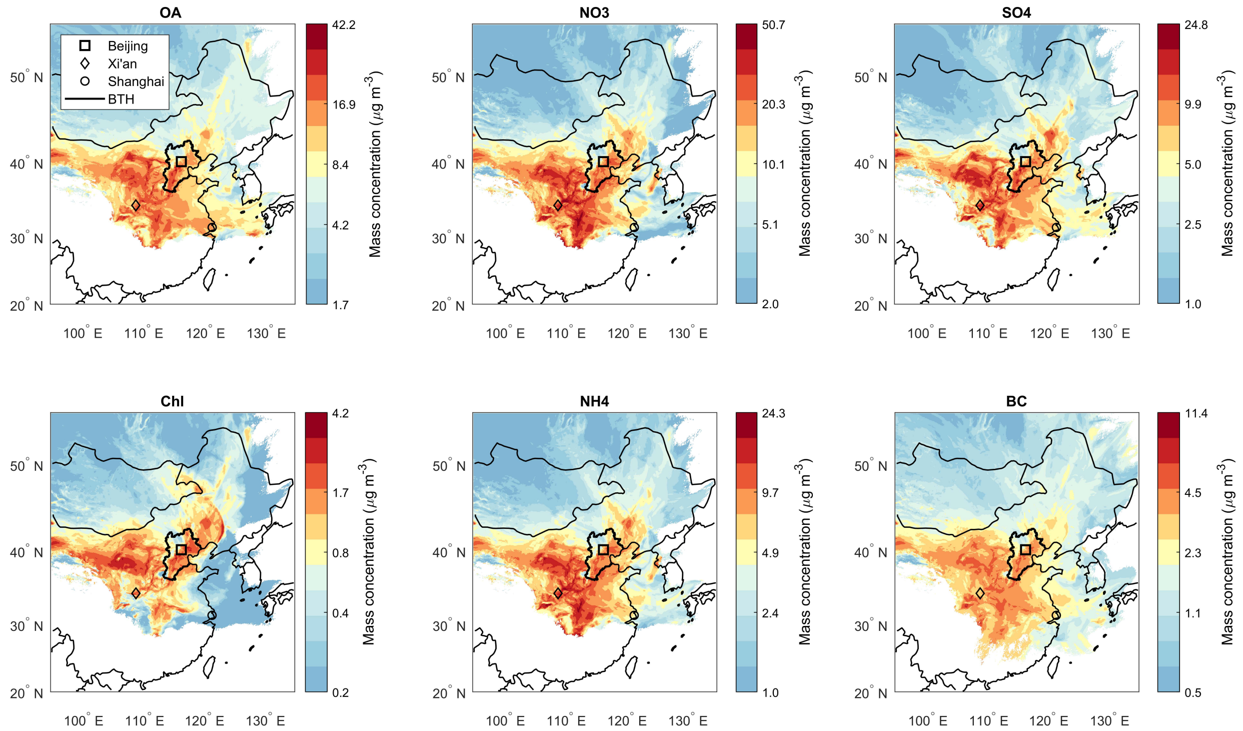

Figure SI 9: 3-day backward dispersion maps of air observed at the Beijing site in contact with the surface, colored by bulk PM2.5 constituent concentration observed at the site. The average for the whole measurement period is shown. High values over specific areas suggest strong precursor emissions in these areas (or within the typical transport route passing over this area). The middle of the colorbar shows the mean value of the compound over the whole observation period and the axis extends to 5 times the mean and 0.2 times the mean in logarithmic scale.

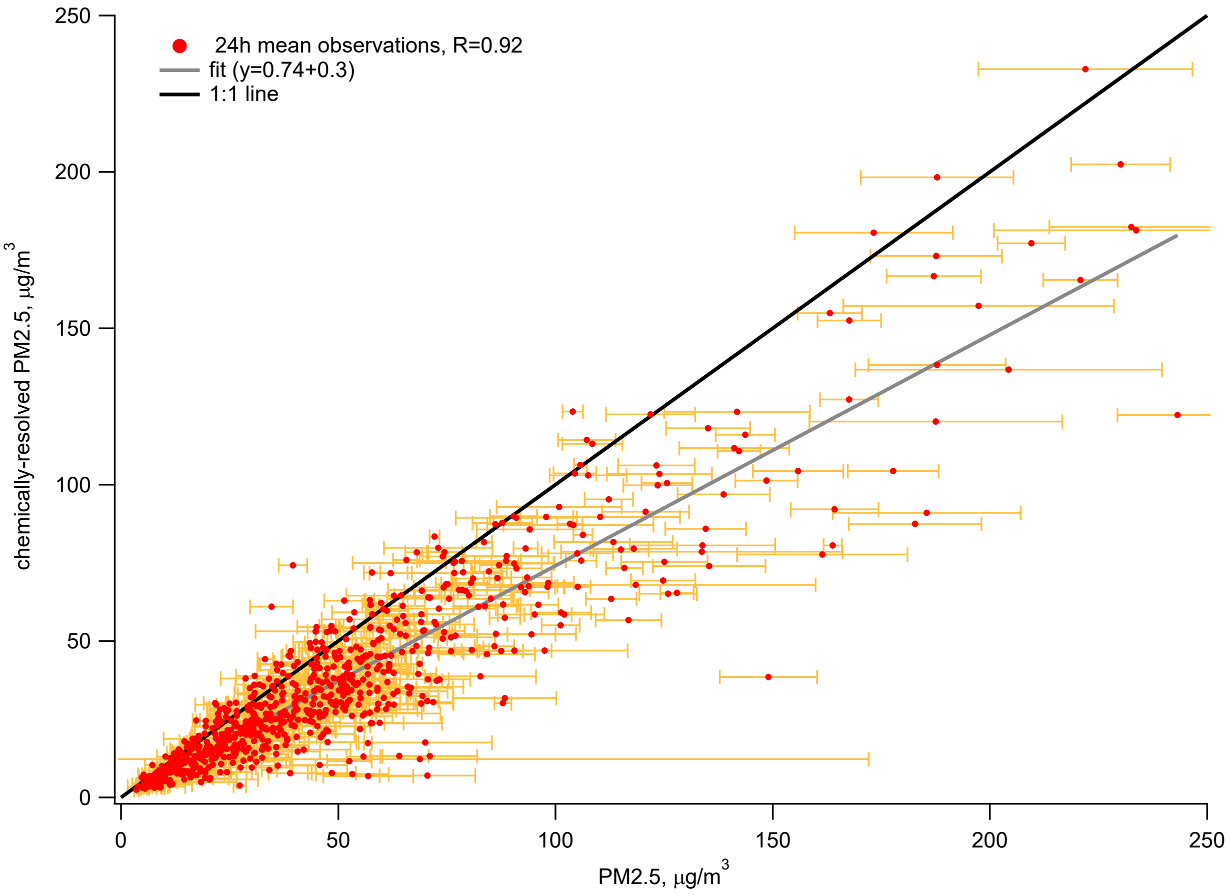

Figure SI 10: Comparison between chemically resolved PM2.5 (non-refractory PM + eBC) and bulk PM2.5 (from surrounding stations, error bars represent the standard deviation in concentration between the surrounding stations).

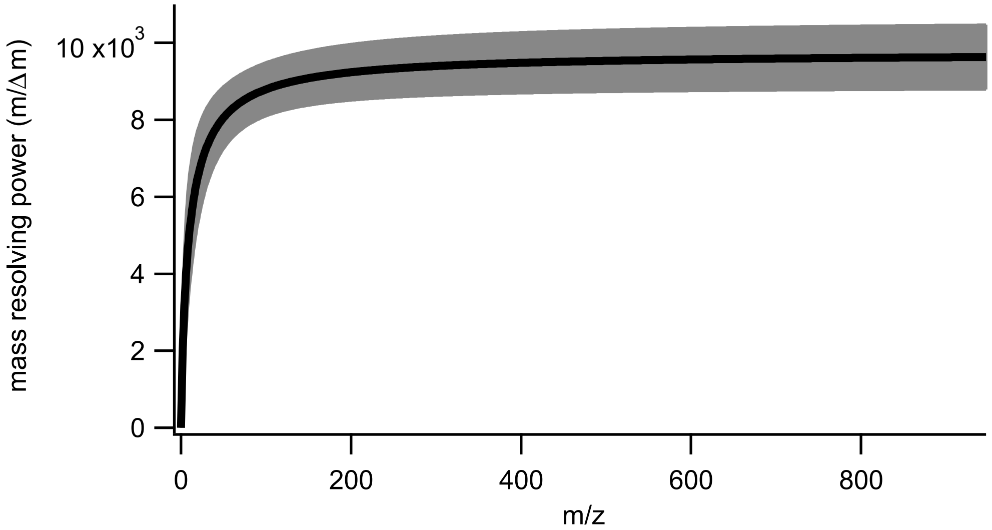

Figure SI 11: Mass resolving power of FIGAERO-CIMS equipped with a LToF mass analyzer (line: average, grey shaded area within standard deviation).

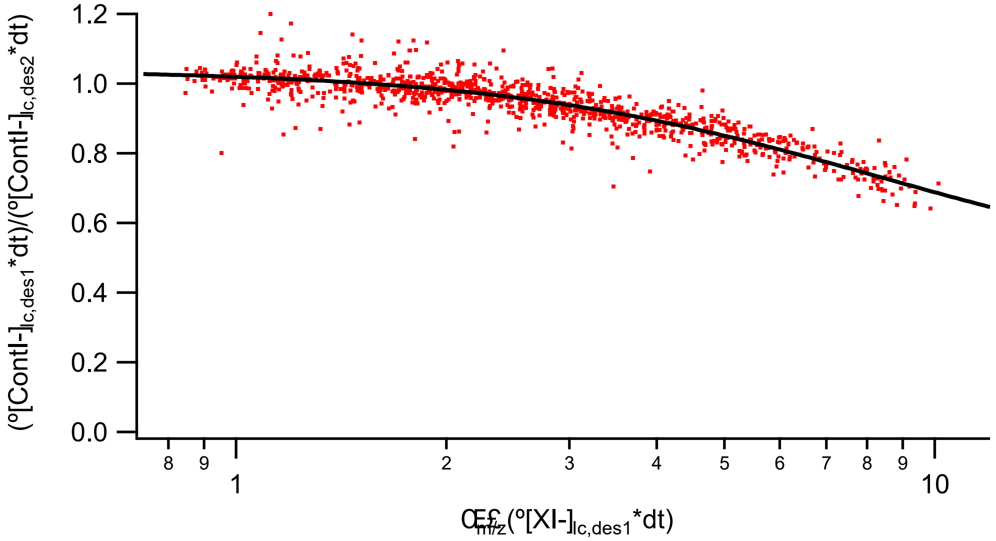

Figure SI 12: Ratio of a contaminant's signal ($[XI-]_{I_c}$) integrated over the entire thermogram during the first compared to the second desorption cycle as a function of the total signal ($[XI-]_{I_c}$) from ions other than the reagent ion.

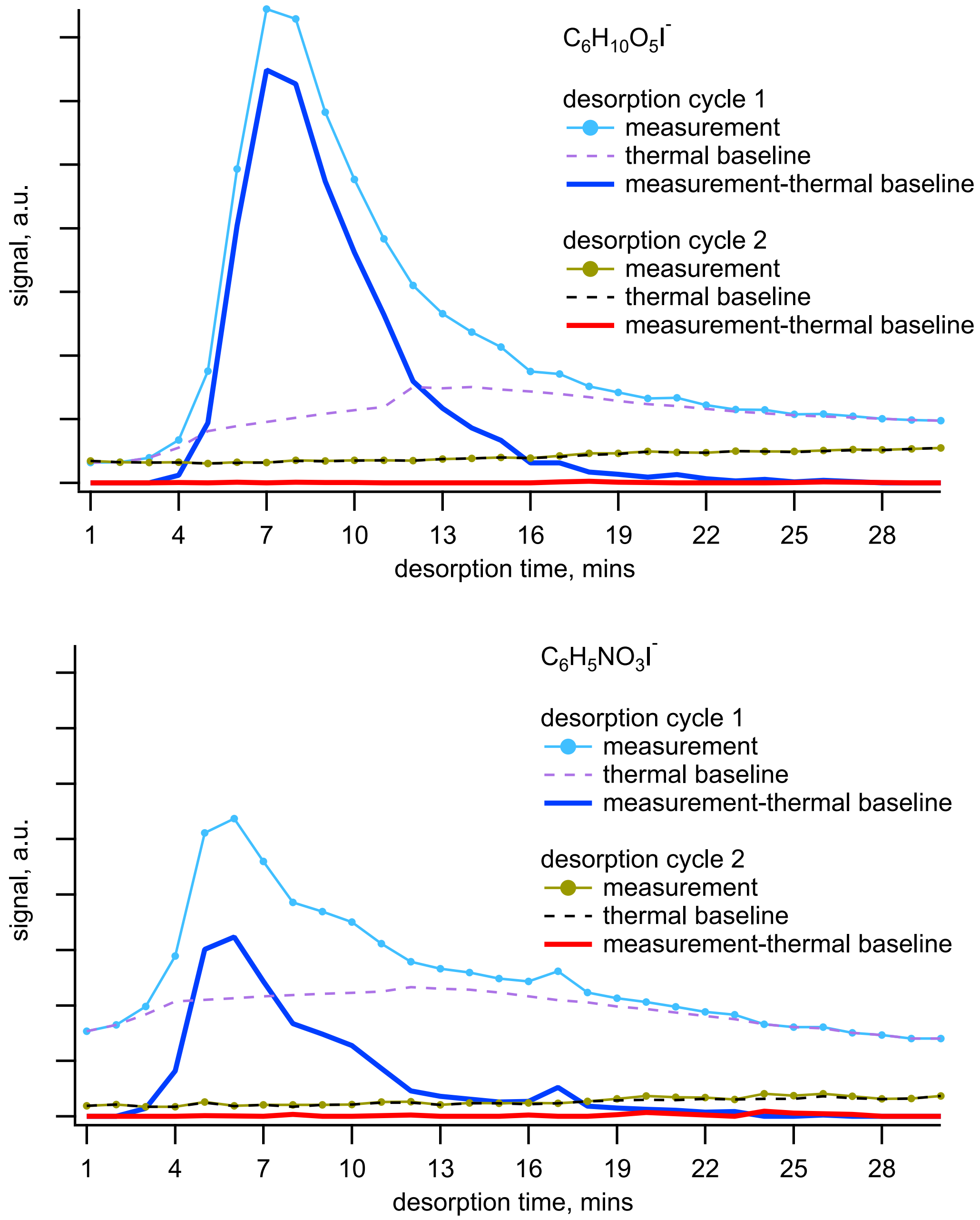

Figure SI 13: Illustration of the thermal baseline computation based on two peaks.

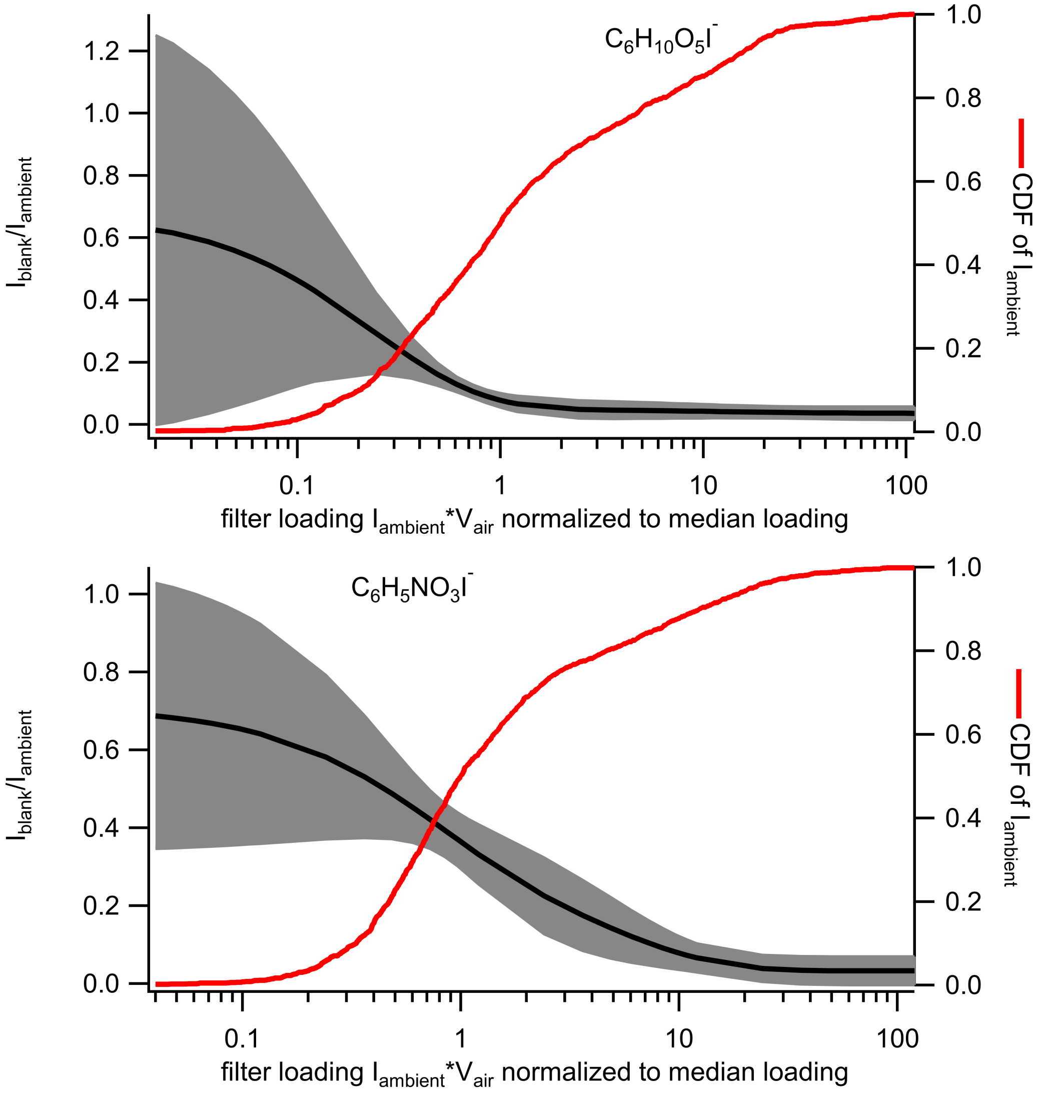

Figure SI 14: Blank parametrization for two example peaks ($C_6H_5NO_3I^-$, $C_6H_{10}O_5I^-$) as $I_{blank}/I_{ambient}$ as a function of the filter loading ($I_{ambient}*V_{air}$) as well as the cumulative distribution function (CDF) of ambient measurements.

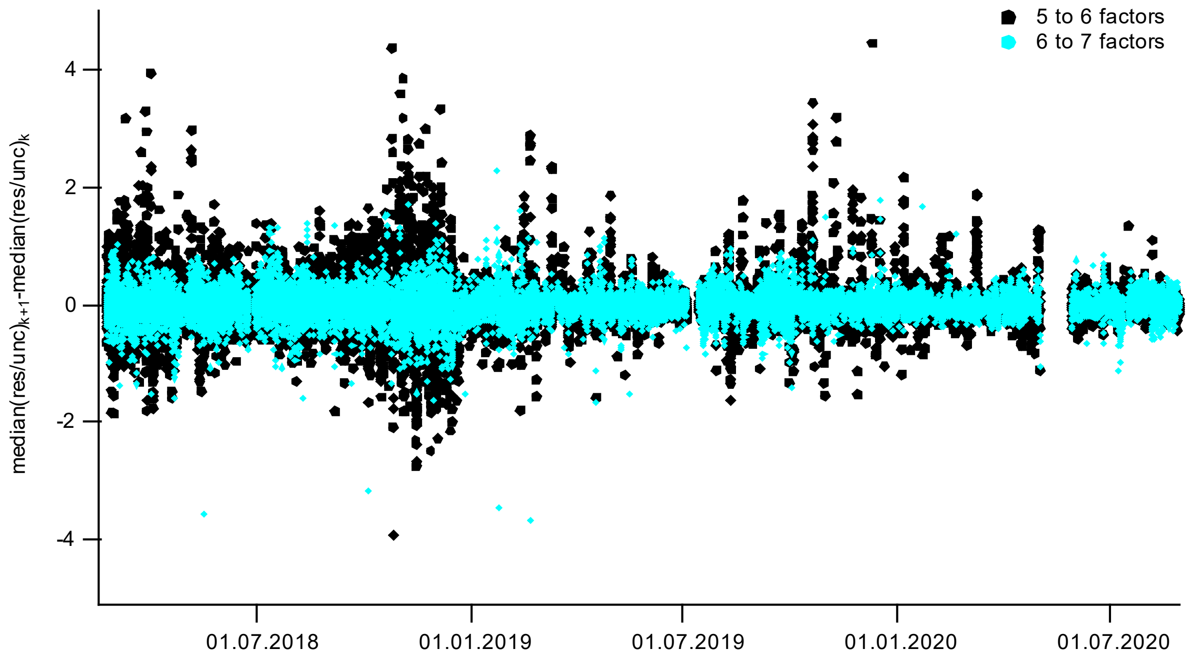

Figure SI 15: Change in time-dependent residual-to-measurement uncertainty ratios as a function of the number of factors).

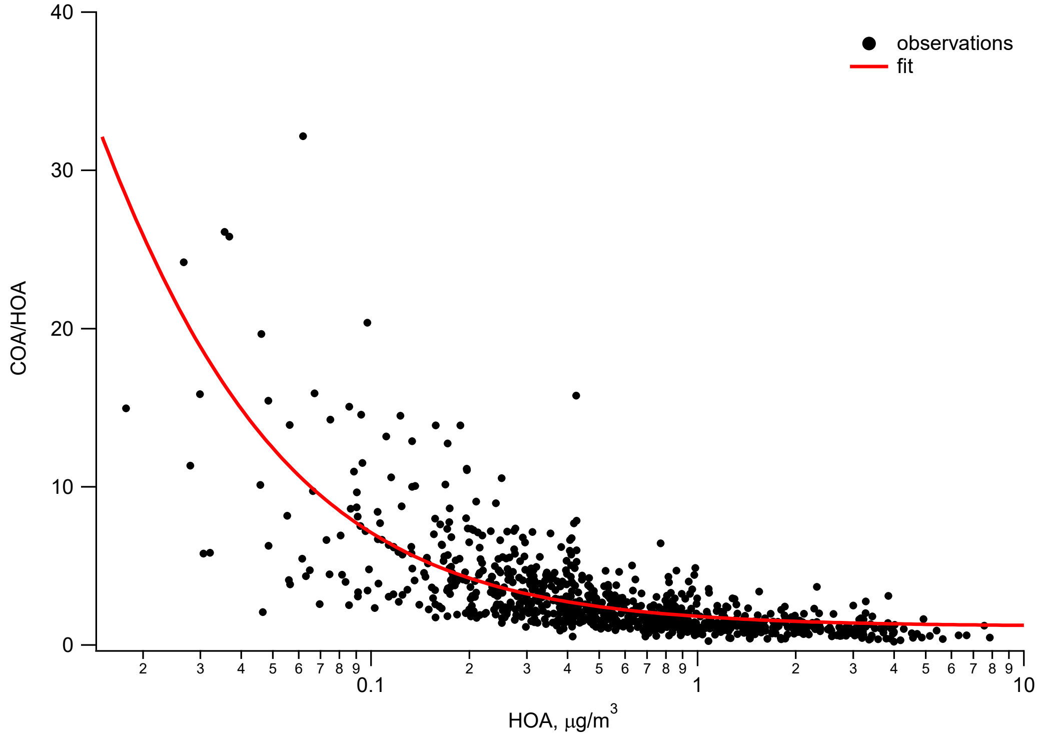

Figure SI 16: Ratio of daily mean concentrations of COA to HOA as a function of HOA (assuming RIEs of 1.4).

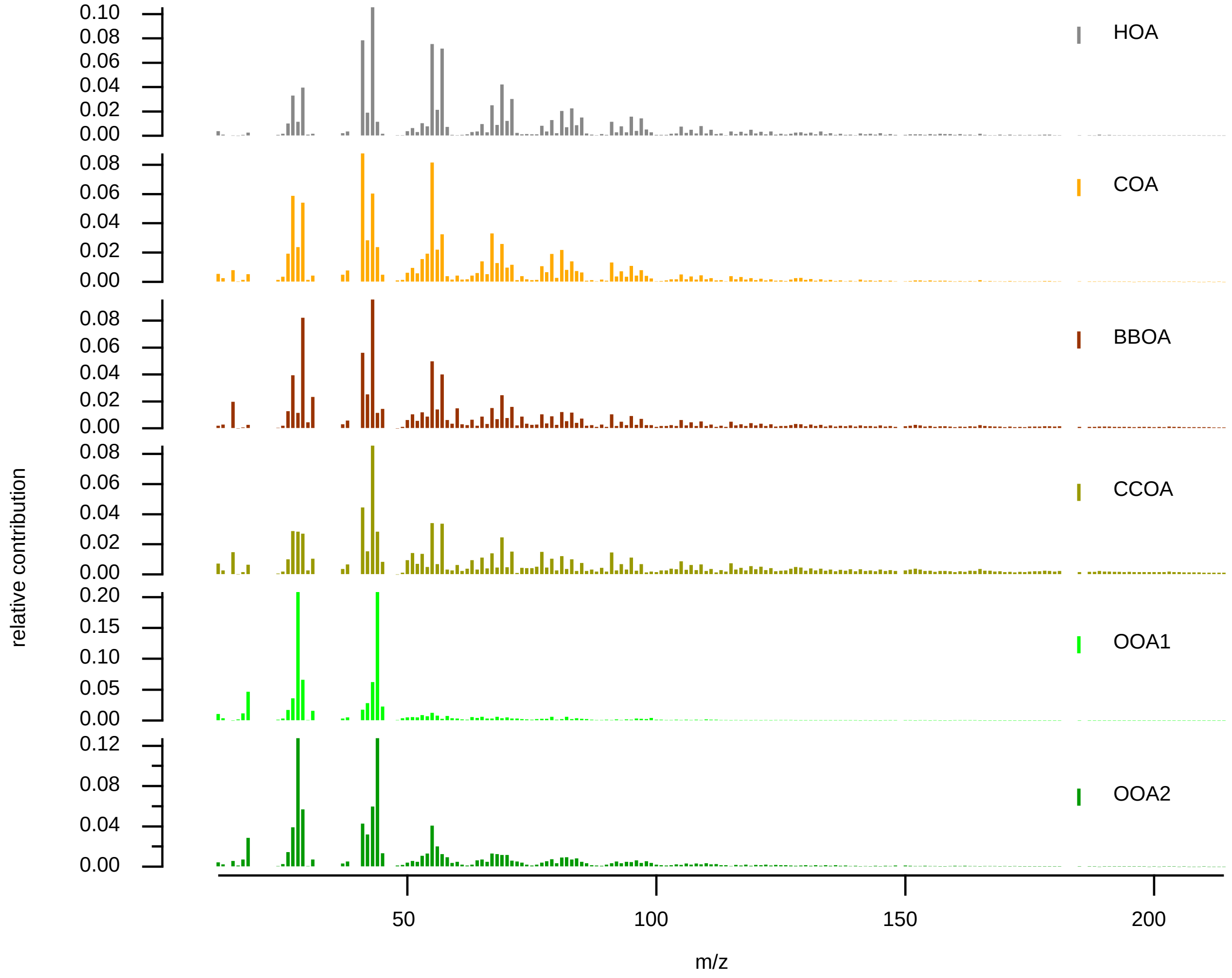

Figure SI 17: Chemical fingerprints of OA components from PMF analysis using ToF-ACSM data.

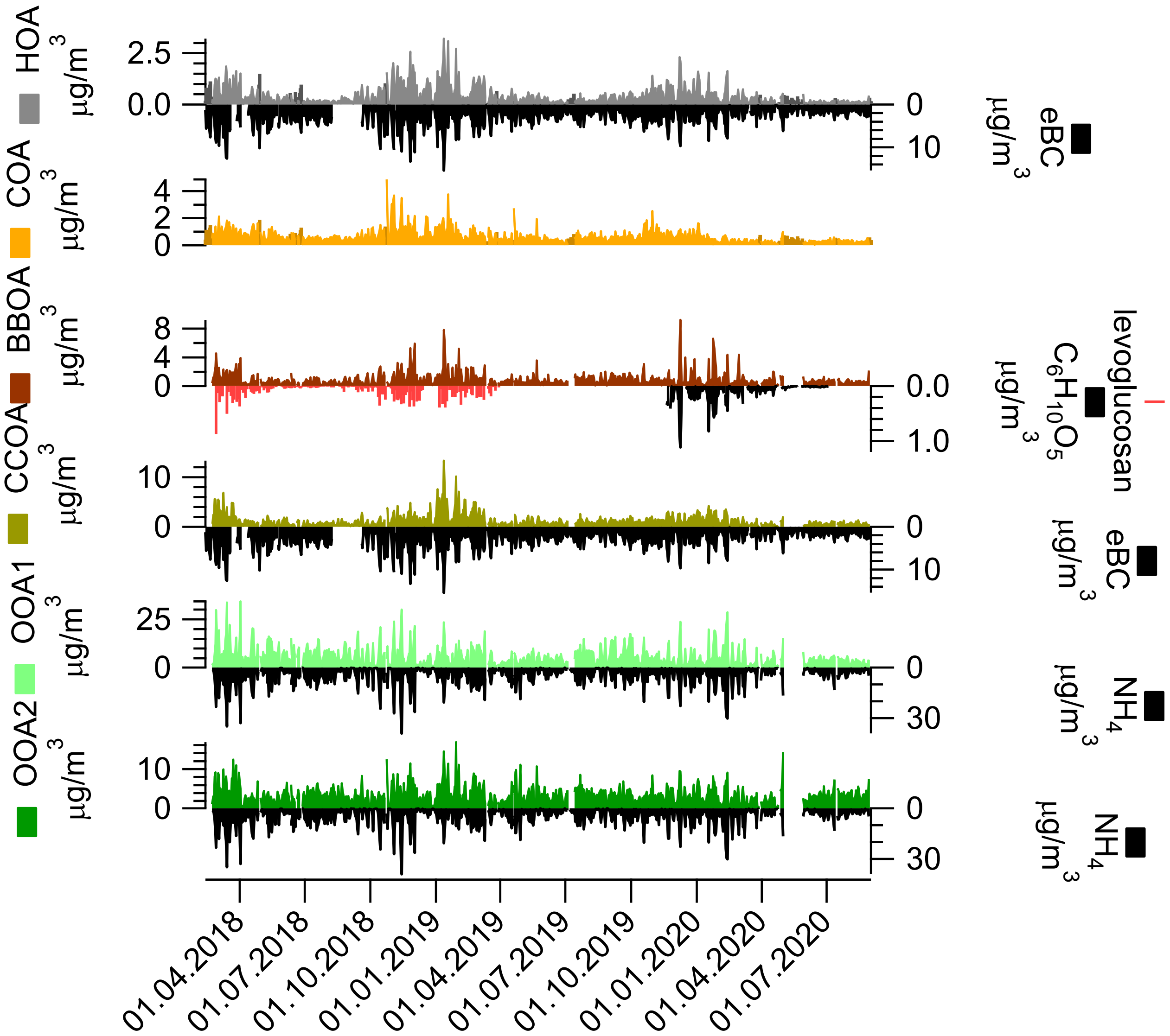

Figure SI 18: Daily mean time series of OA components (HOA, COA, BBOA, CCOA, OOA1, OOA2) and selected PM constituents (eBC, levoglucosan, NH_4). Some of the PM constituents (eBC, NH_4) are displayed twice to facilitate comparison to the OA components.

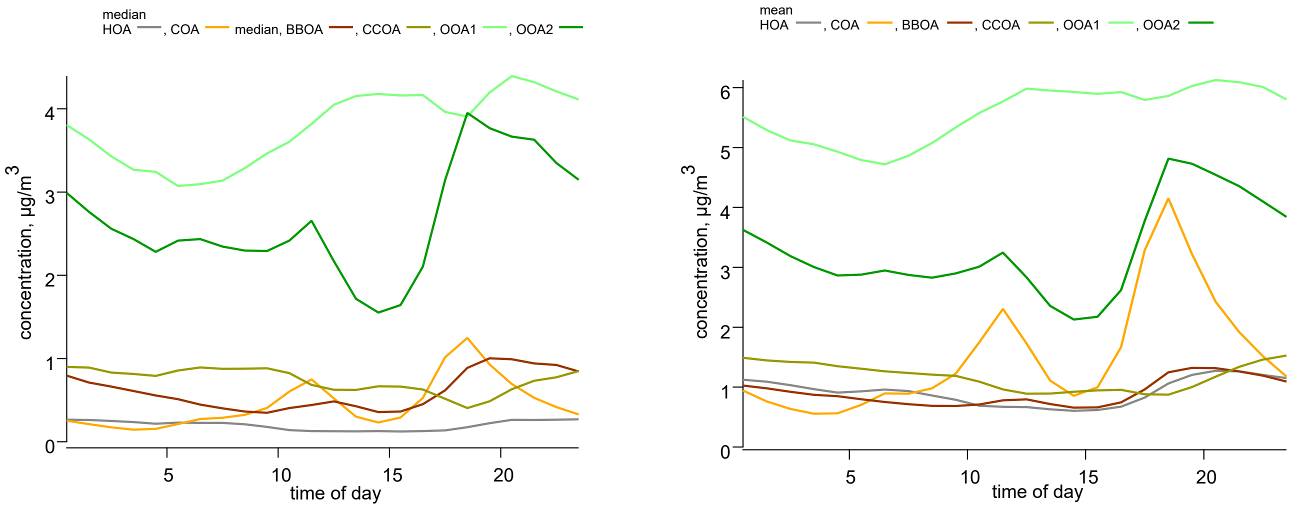

Figure SI 19: Diurnal concentration variation of ACSM OA components (HOA, COA, BBOA, CCOA, OOA1, OOA2).

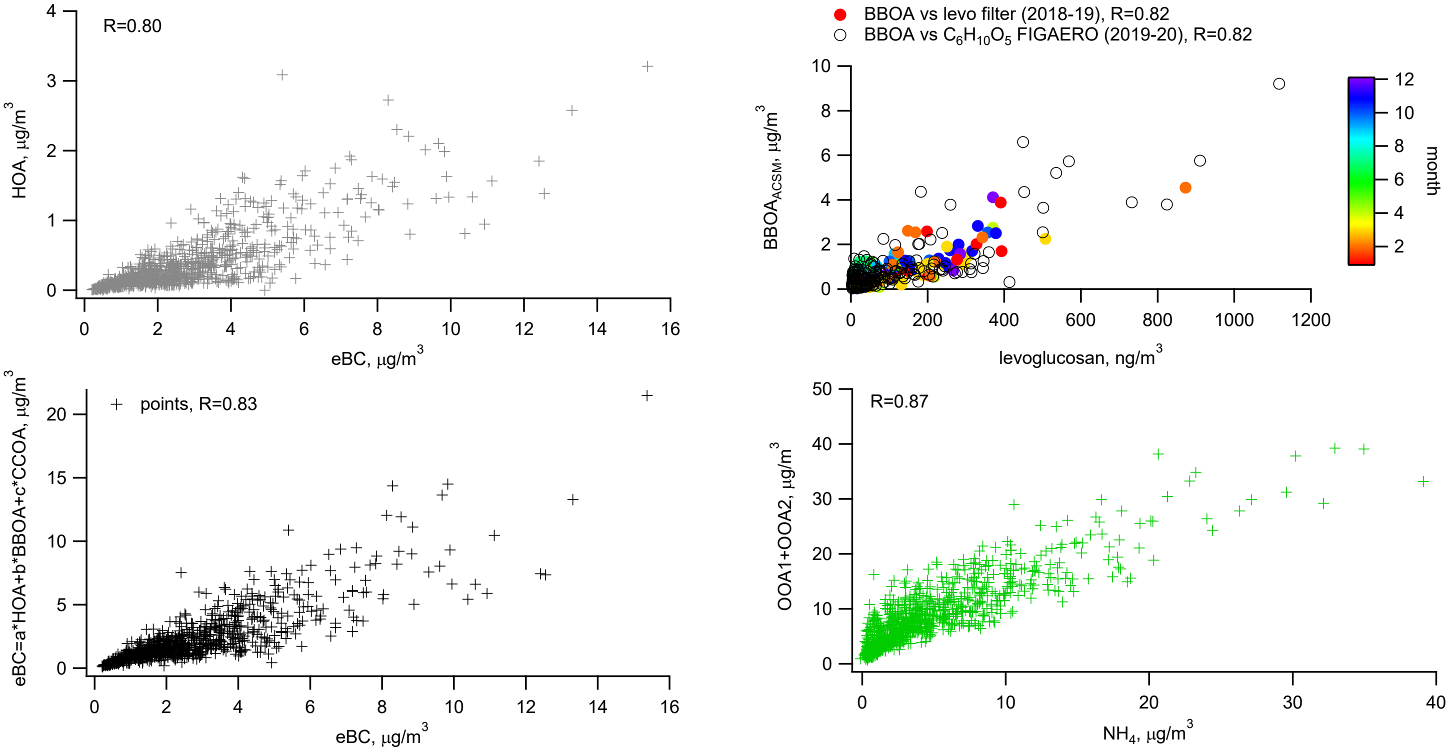

Figure SI 20: Comparison between selected OA components to markers (daily mean concentrations).

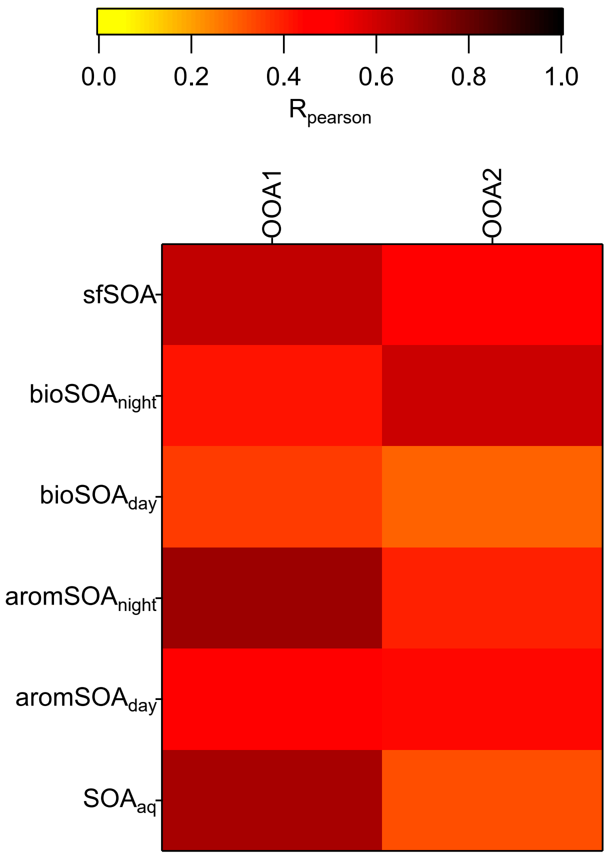

Figure SI 21: SOA sources correlation with ACSM OOA1 and ACSM OOA2

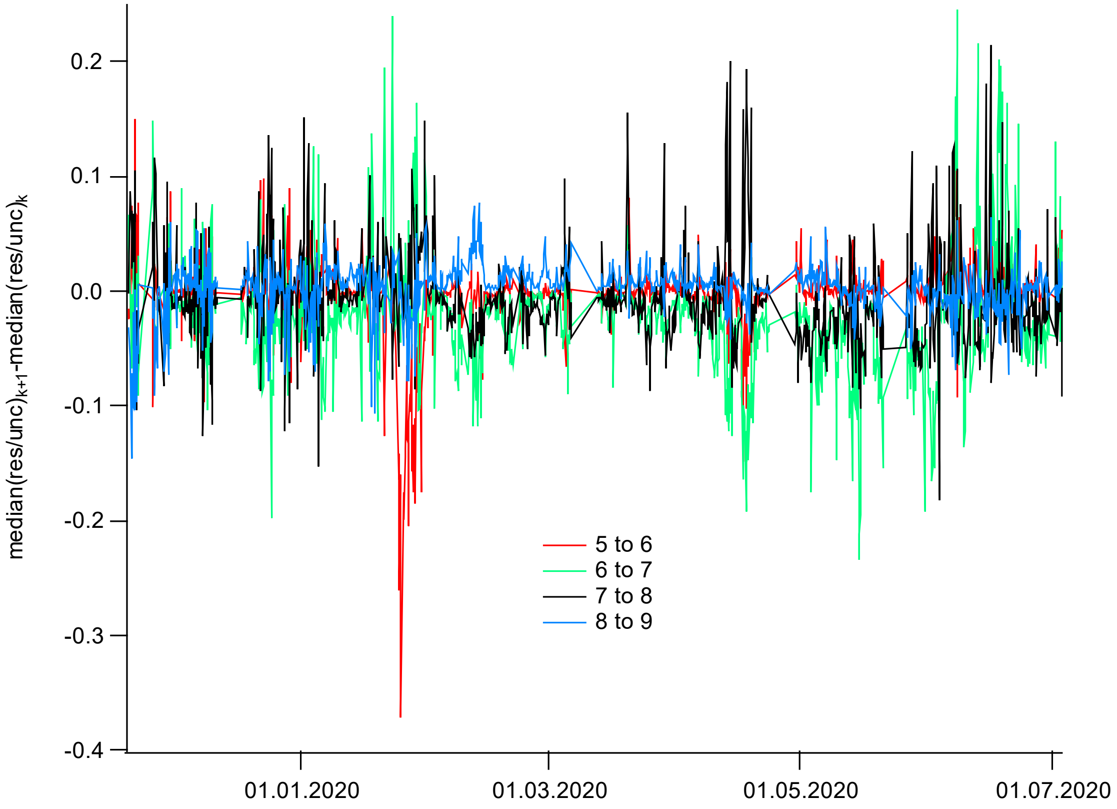

Figure SI 22: Change in the time-dependent median residual-to-measurement uncertainty ratio as a function of the number of factors (5 to 9 factors).

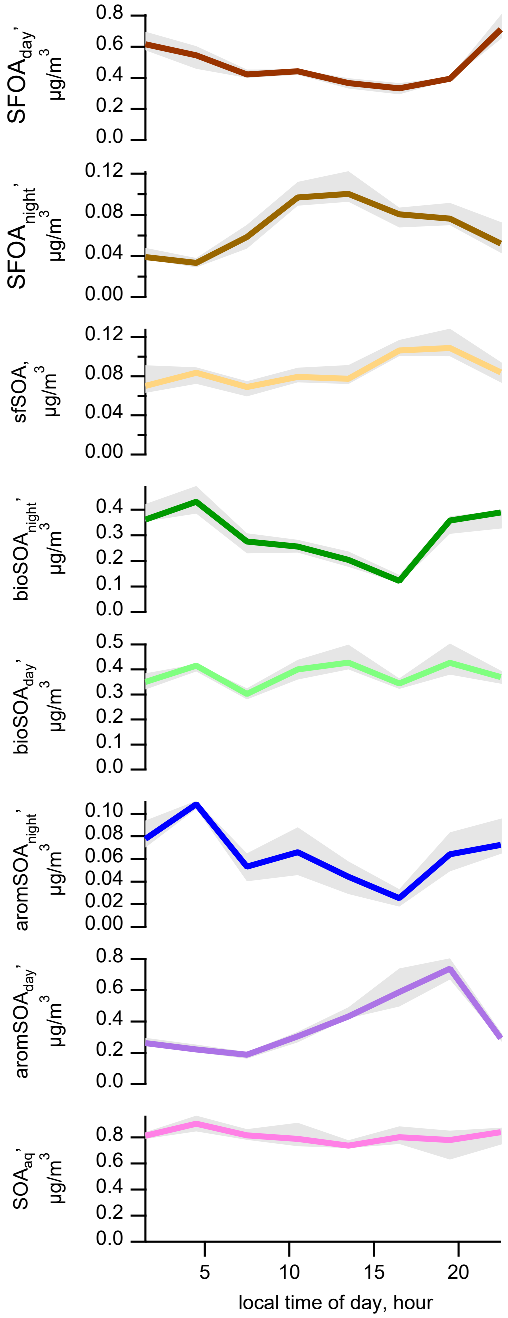

Figure SI 23: Diurnal variation of all FIGAERO-CIMS types shown as median diel cycle. The uncertainty range shown as grey shaded area represents the interquartile range (25 percentile to 75 percentile) from a bootstrapping analysis (1000 runs), i.e. resampling with replacement, of all measurement points at a given time of day.

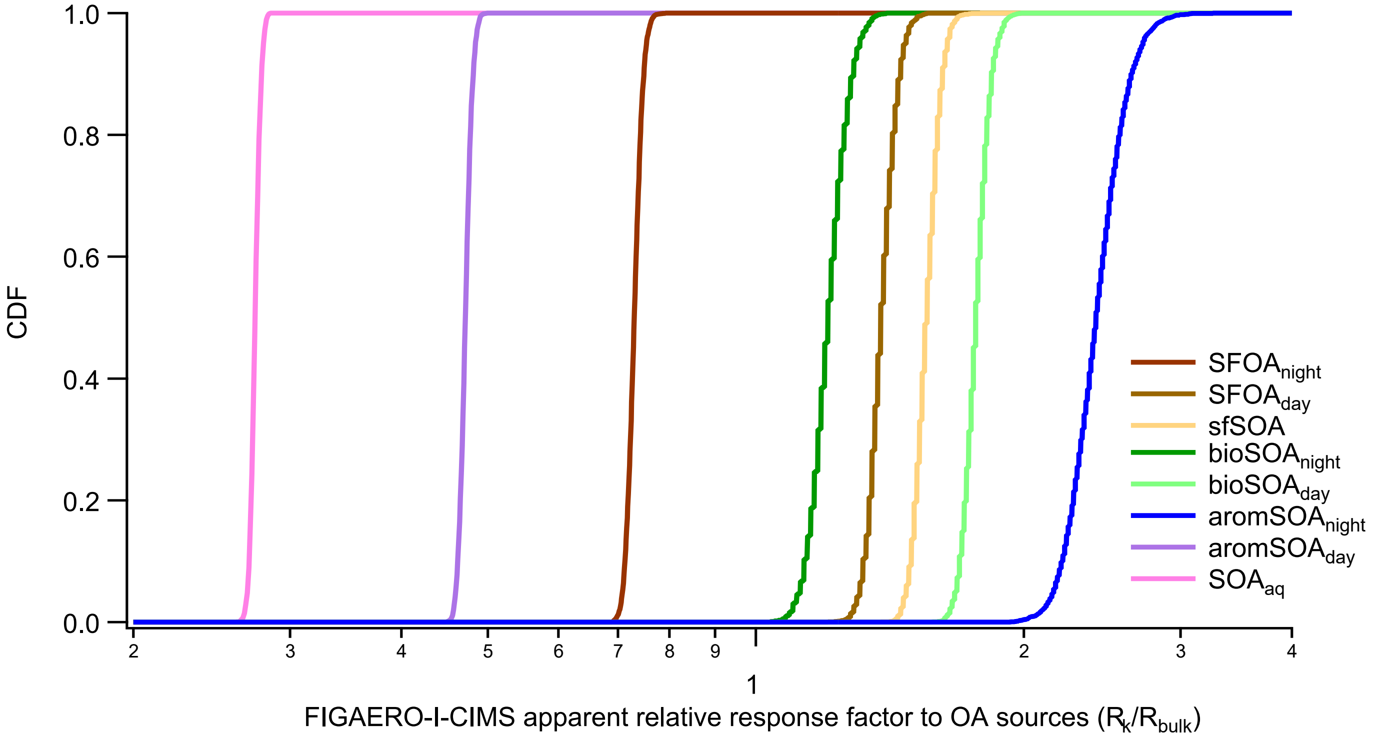

Figure SI 24: FIGAERO-CIMS relative response factors and their MLR-fitting uncertainty to different OA factors are visualized as cumulative distribution functions. To that purpose the factor-specific FIGAERO-CIMS response factors were normalized to the bulk OA response factor (relative response factors are: SFOAnight 0.731±0.014, SFOAday 1.386±0.050, sfSOA 1.556±0.051, bioSOAnight 1.209±0.058, bioSOAday 1.769±0.058, aromSOAnight 2.421±0.174, aromSOAday 0.472±0.007, SOAaq 0.274±0.003).

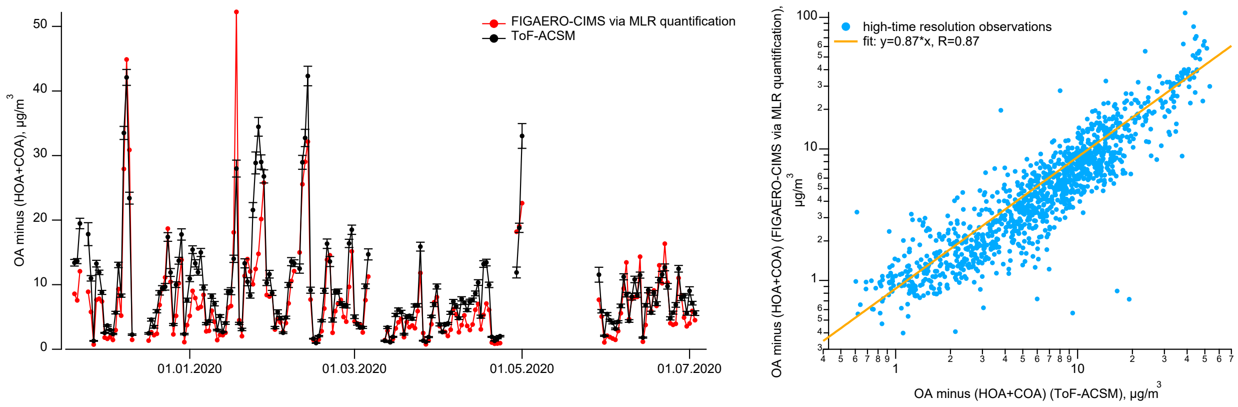

Figure SI 25: Comparison between FIGAERO-CIMS and ToF-ACSM: a) Daily mean concentration timeseries from FIGAERO-CIMS and ToF-ACSM, b) Scatterplot of high-time resolution concentration timeseries from FIGAERO-CIMS and ToF-ACSM.

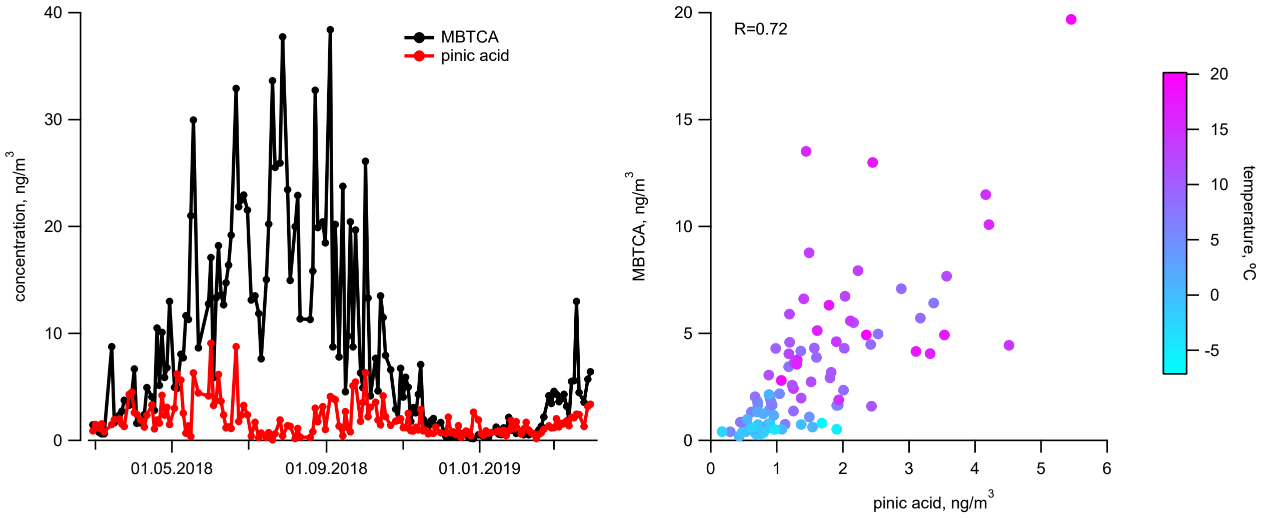

Figure SI 26: a) Particle-phase MBTCA and pinic acid seasonality, b) comparison of particle-phase MBTCA and pinic acid concentrations including days with a daily average temperature up to 20 C. At higher temperatures the the correlation deteriorates because of partitioning: Pinic acid preferentially resides in the gas-phase while MBTCA is less affected.

References

1 Li, M. *et al.* MIX: a mosaic Asian anthropogenic emission inventory under the international collaboration framework of the MICS-Asia and HTAP. *Atmos. Chem. Phys.* **17**, 935-963 (2017). https://doi.org:10.5194/acp-17-935-2017
